# Supplementary material for: In vivo base editing reduces liver cysts in autosomal dominant polycystic kidney disease
Source: Mol Ther. 2025 Aug 20;33(11):5373–82. doi: 10.1016/j.ymthe.2025.08.026 (PMC12628146; doi:10.1016/j.ymthe.2025.08.026)
Supplement: Document S1. Figures S1–S3 and Tables S1 and S2 [file mmc1.pdf]

YMTHE, Volume *33*

Supplemental Information

In vivo base editing reduces liver cysts in autosomal dominant polycystic kidney disease

Antonia Ibel, Rishi Bhardwaj, Duygu Elif Yilmaz, Shuhan Kong, Sarah Wendlinger, Carlos Cordero, Dimitra Papaioannou, Maria Papazian, Ria Schönauer, Qiyao Meng, Kai-Uwe Eckardt, Fatima Hassan, Isabel Volpe, Verena Klämbt, Jan Halbritter, Sorin Fedeles, Matteus Krappitz, and Michael M. Kaminski

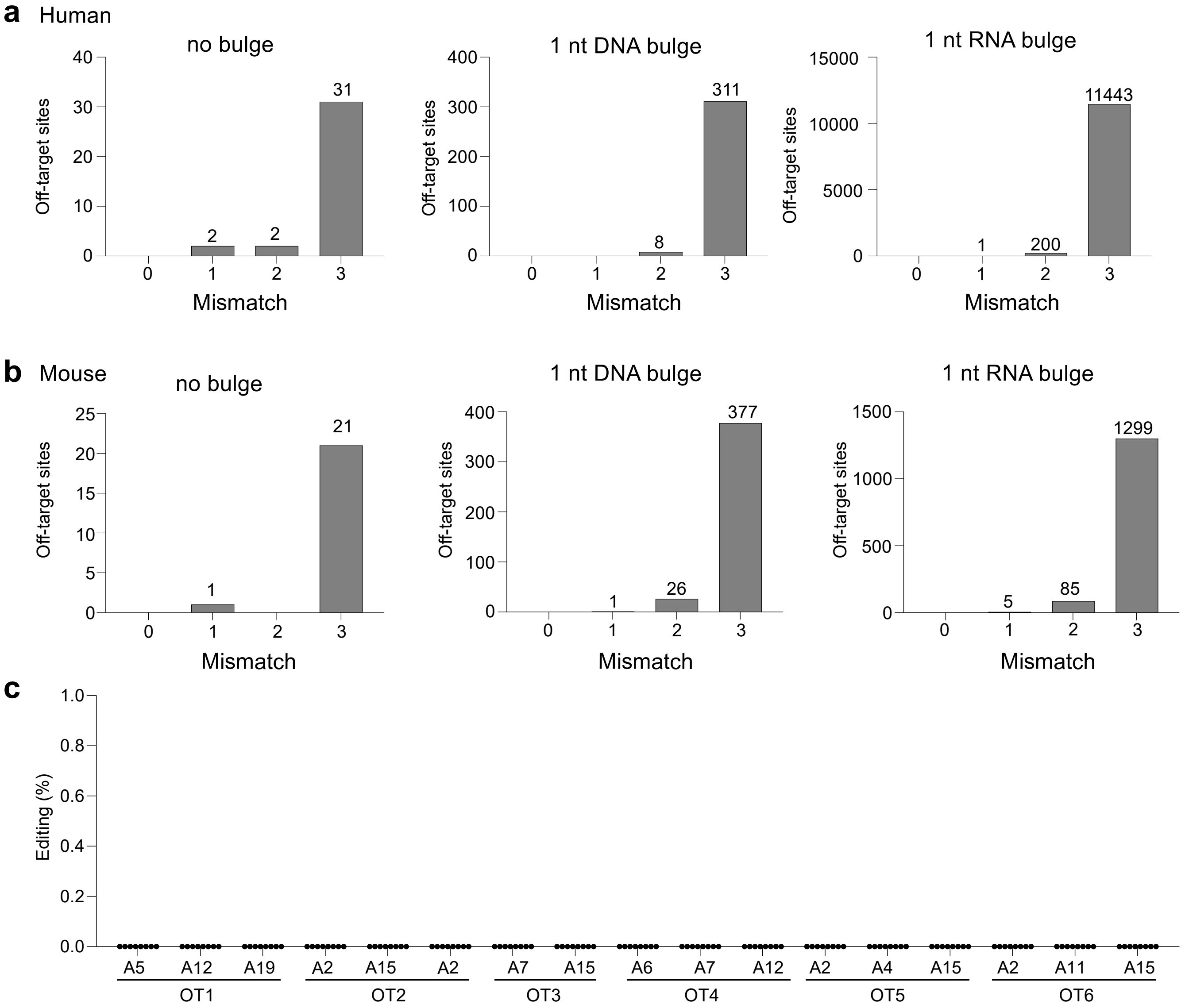

Figure S1: Off-target analysis for RW correcting human and mouse sgRNAs. a, Number of CasOFFinder annotated putative off-target sites in the human genome for sgRNA_20 (for SpCas9 and a NGG PAM). Predicted off-target sites for one and two mismatch sites (without bulges) were identified within *PKD1* pseudogenes**. b,** Number of CasOFFinder annotated putative off-target sites in the mouse genome for sgRNA_45 (for SpCas9 and a NGG PAM). The one site predicted for one mismatch (no bulge) marks the target site. **c,** *In vivo* editing efficiency (y-axis) for the top 6 predicted mouse off-target sites (OT) as determined by targeted amplicon sequencing of gDNA isolated from liver tissue at all possible adenines (A).

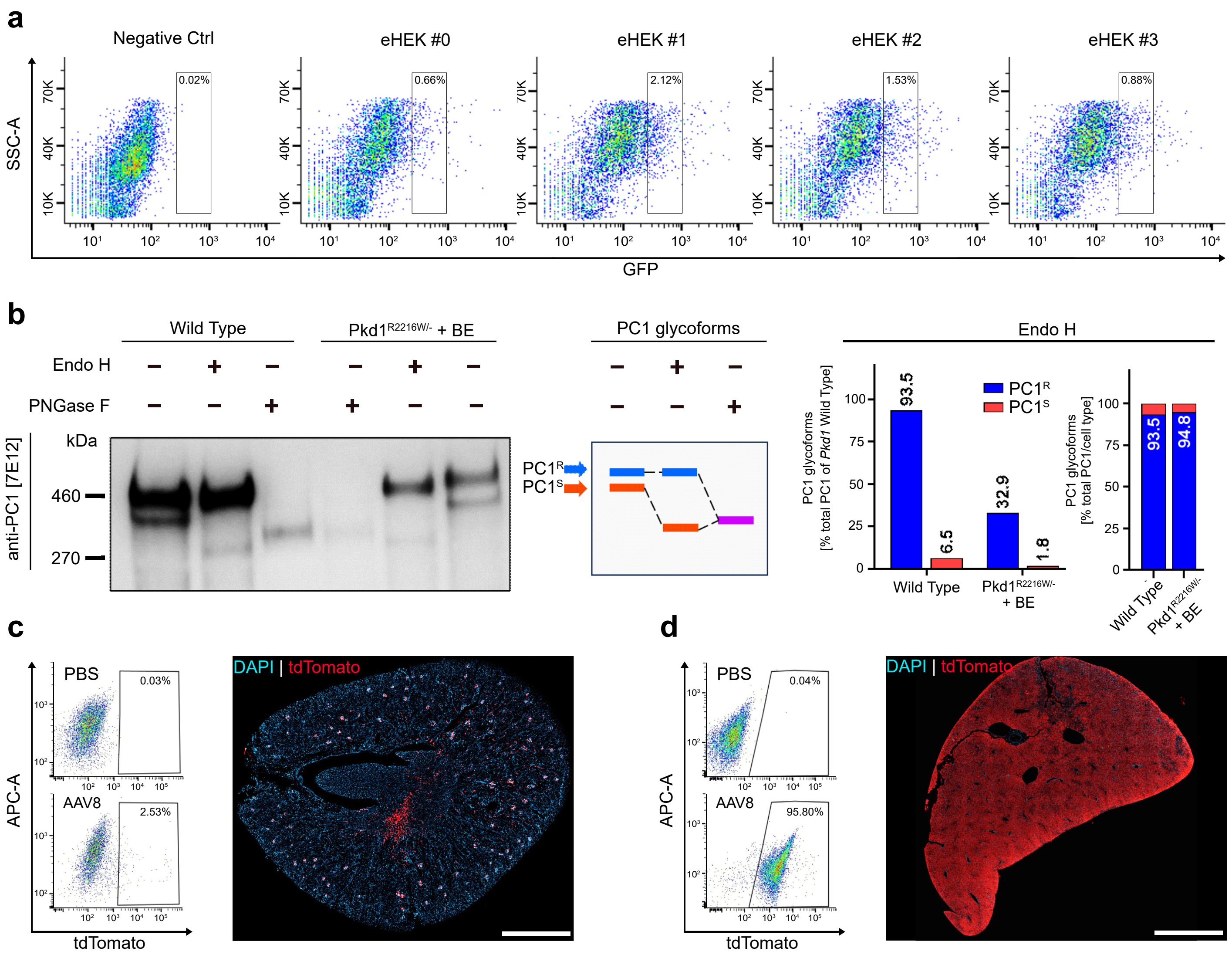

Figure S2: Quality control for engineered HEK293T cell lines and RTEC editing and assessment of *in vivo* AAV8 transduction efficiency in the kidney and liver. a, FACS plots illustrating the gating strategy used for sorting the four engineered HEK293T cell lines (eHEK #0-#3). Transfection and sorting were performed on the same day, applying identical gates to select for cells with medium-to-low expression levels to ensure consistent and low copy number integration across all four lines. **b,** Deglycosylation analysis of PC1 in base edited and control RTEC cells reveals comparable amounts of mature Endo H-resistant ($PC1^R$; blue) and immature Endo H-sensitive ($PC1^S$, red) glycoforms. PNGase F served as a positive control for complete deglycosylation (violet). **c,** Systemic AAV8-Cre delivery ($1x10^{13}$VG/kg) in Ai14 reporter mice show limited transduction in the kidney. Left panel: flow cytometry analysis of tdTomato expresion in dissociated kidney cells. Top left plot shows kidney cells from a PBS-injected control mouse, while the bottom left plot shows kidney cells from an AAV8-Cre–injected mouse. Right panel: representative immunofluorescence image of a coronal kidney section from an AAV8-Cre–injected mouse. tdTomato expression is shown in red; nuclei are counterstained with DAPI (turquoise). Scale bar = 1000 µm. **d,** Systemic AAV8-Cre delivery ($1x10^{13}$ VG/kg) in Ai14 reporter mice show successful transduction in the liver. Left panel: flow cytometry analysis of tdTomato expression in dissociated liver cells. Top left plot shows liver cells from a PBS-injected control mouse; the bottom left plot shows liver cells from an AAV8-Cre–injected mouse. Right panel: representative immunofluorescence image of a liver section from an AAV8-Cre–injected mouse. tdTomato expression is shown in red; nuclei are counterstained with DAPI (turquoise). Scale bar = 1000 µm.

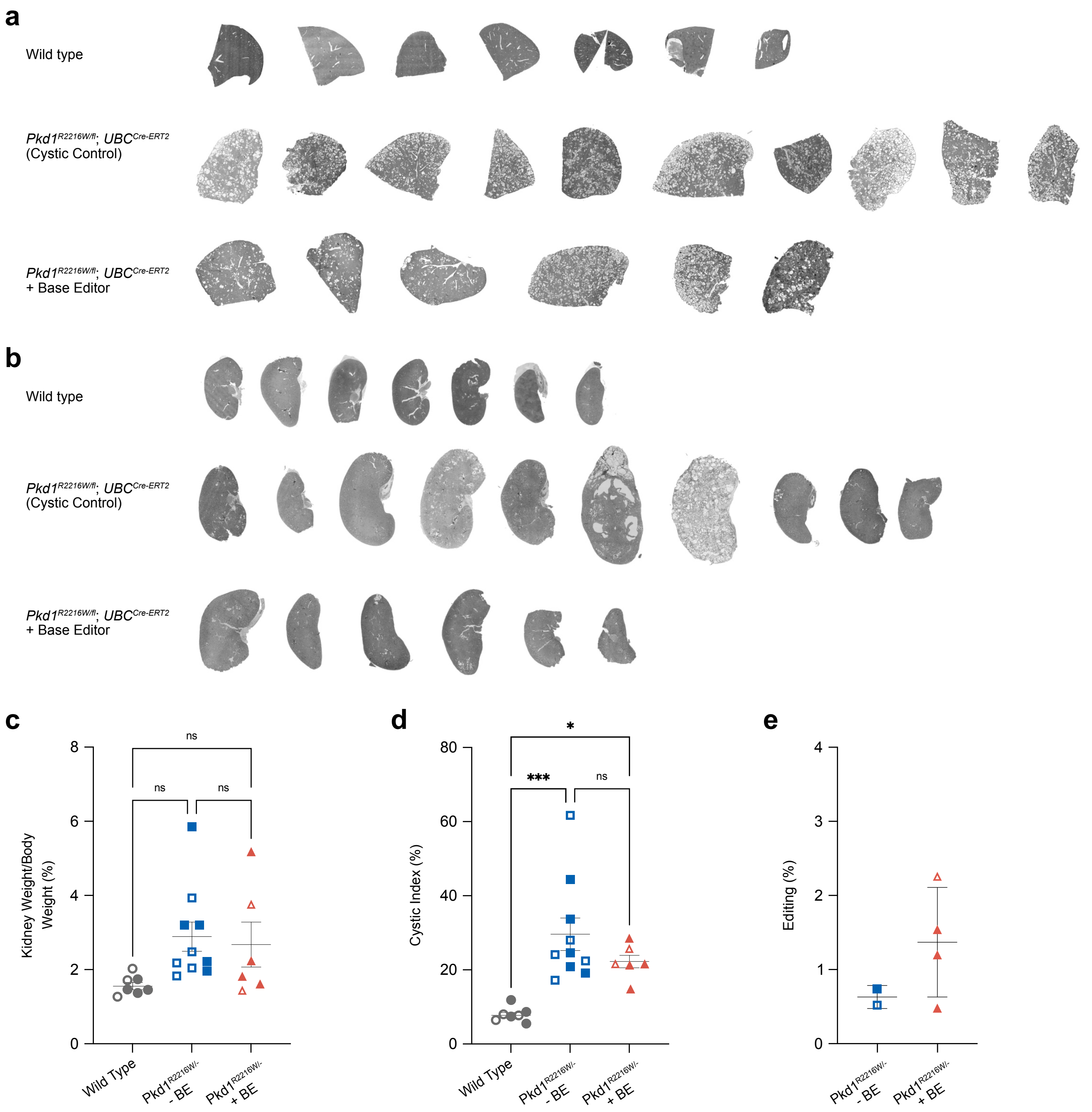

Figure S3: *In vivo* base editing shows no influence on kidney phenotype in the RW knock-in mouse model. a, Microscopic overview images of liver tissue from the indicated conditions from all animals. **b,** Microscopic overview images of kidney tissue from the indicated conditions from all animals. **c, d,** Quantification of kidney-to-body weight ratios (**c**) and kidney cystic indices (**d**) between wild-type mice (n=7), untreated RW knock-in mice (n=10) and base editor (BE) treated RW knock-in mice (n=6). **e,** Quantification of editing efficiency in gDNA isolated from the kidney of the untreated RW knock-in mice (n=2) and base editor (BE) treated RW knock-in mice (n=4). **c, d, e,** Data points represent independent biological replicates with means ± SD, $***p < 0.001$, $*p < 0.05$, ns: not significant. Filled data points indicate male mice, hollow once female mice.

Table S1: Primer sequences for targeted amplicon sequencing (TAS) and long-range PCR (LR-PCR). For TAS, PCR-1 was used for target locus–specific amplification and PCR-2 for addition of barcodes and Illumina adapter sequences. All sequences are shown in the 5′→3′ orientation.

Table S1

**Primer Name**	**Primer Sequence (5'-3')**	**Used for**
16SrRNA_63f fwd/fwd_TAS	TCGTCGGCAGCGTCAGATGTGTATAAGAGACAGCAGGCCTAACACATGCAAGTC	PCR-1
16SrRNA_1387f rev/rev_TAS	GTCTCGTGGGCTCGGAGATGTGTATAAGAGACAGGCCTTGTACACTCCGCCC	PCR-1
16SrRNA_1387r rev/fwd_TAS	TCGTCGGCAGCGTCAGATGTGTATAAGAGACAGGGGCGGAGTGTACAAGGC	PCR-1
AmpR rev_TAS	GTCTCGTGGGCTCGGAGATGTGTATAAGAGACAGGCTATGTGGCGCGGTATTAT	PCR-1
AmpR fwd_TAS	TCGTCGGCAGCGTCAGATGTGTATAAGAGACAGATAATACCGCGCCACATAGC	PCR-1
LucNrev rev_TAS	GTCTCGTGGGCTCGGAGATGTGTATAAGAGACAGGGAGAGCAACTGCATAAGG	PCR-1
LucNrev fwd_TAS	TCGTCGGCAGCGTCAGATGTGTATAAGAGACAGCCTTATGCAGTTGCTCTCC	PCR-1
Partial-P5 rev_TAS	GTCTCGTGGGCTCGGAGATGTGTATAAGAGACAGTCTCGGTGGTCGCCGTATCATT	PCR-1
Partial-P5 fwd_TAS	TCGTCGGCAGCGTCAGATGTGTATAAGAGACAGAATGATACGGCGACCACCGAGA	PCR-1
pUC19-fwd/rev_TAS	GTCTCGTGGGCTCGGAGATGTGTATAAGAGACAGCTTAGACGTCAGGTGGCACTT	PCR-1
pUC19-fwd/fwd_TAS	TCGTCGGCAGCGTCAGATGTGTATAAGAGACAGAAGTGCCACCTGACGTCTAAG	PCR-1
TSO enrichment rev_TAS	GTCTCGTGGGCTCGGAGATGTGTATAAGAGACAGAAGCAGTGGTATCAACGCAGAGT	PCR-1
TSO enrichment fwd_TAS	TCGTCGGCAGCGTCAGATGTGTATAAGAGACAGACTCTGCGTTGATACCACTGCTT	PCR-1
CMV Forward rev_TAS	GTCTCGTGGGCTCGGAGATGTGTATAAGAGACAGCACGCCTACCGCCCATTTGCG	PCR-1
CMV Forward fwd_TAS	TCGTCGGCAGCGTCAGATGTGTATAAGAGACAGCGCAAATGGGCGGTAGGCGTG	PCR-1
VR Primer rev_TAS	GTCTCGTGGGCTCGGAGATGTGTATAAGAGACAGGCTCACTCAAAGGCGGTAAT	PCR-1
VR Primer fwd_TAS	TCGTCGGCAGCGTCAGATGTGTATAAGAGACAGATTACCGCCTTTGAGTGAGC	PCR-1
oAI411_LucNrev_fwd	TCGTCGGCAGCGTCAGATGTGTATAAGAGACAGTATGCAGTTGCTCTCCGCACT	PCR-1
oAI411_AmpR_rev	GTCTCGTGGGCTCGGAGATGTGTATAAGAGACAGTATGTGGCGCGGTATTATCGTG	PCR-1
oAI411_AmpR_fwd	TCGTCGGCAGCGTCAGATGTGTATAAGAGACAGCACGATAATACCGCGCCACATA	PCR-1
oAI411_16S rRNA Primer 1387r_rev	GTCTCGTGGGCTCGGAGATGTGTATAAGAGACAGTTGTACACTCCGCCCACCGT	PCR-1
oAI411_16S rRNA Primer 1387r_fwd	TCGTCGGCAGCGTCAGATGTGTATAAGAGACAGACGGTGGGCGGAGTGTACAA	PCR-1
oAI411_16S rRNA Primer 63f_rev	GTCTCGTGGGCTCGGAGATGTGTATAAGAGACAGGCATGTGTTAGGCCTGTCCTG	PCR-1
oAI412_Tso_Enrich_fwd	TCGTCGGCAGCGTCAGATGTGTATAAGAGACAGGTTGATACCACTGCTTCACG	PCR-1
oAI412_pUC19_rev	GTCTCGTGGGCTCGGAGATGTGTATAAGAGACAGTTAGACGTCAGGTGGCACTTCT	PCR-1
oAI412_LucNrev_fwd	TCGTCGGCAGCGTCAGATGTGTATAAGAGACAGCCTGGCTGCCTTATGCAGTT	PCR-1
oAI412_AmpR_rev	GTCTCGTGGGCTCGGAGATGTGTATAAGAGACAGTCAGCCGTGAGCTATGTGG	PCR-1
oAI412_AmpR_fwd	TCGTCGGCAGCGTCAGATGTGTATAAGAGACAGCCACATAGCTCACGGCTGA	PCR-1
oAI412_16S rRNA Primer 1387r_rev	GTCTCGTGGGCTCGGAGATGTGTATAAGAGACAGGCTGTGCCTTGTACACTCCG	PCR-1
oAI412_16S rRNA Primer 1387r_fwd	TCGTCGGCAGCGTCAGATGTGTATAAGAGACAGCGGAGTGTACAAGGCACAGC	PCR-1
oAI412_16S rRNA Primer 63f_rev	GTCTCGTGGGCTCGGAGATGTGTATAAGAGACAGTGTGTTAGGCCTGACCCCAC	PCR-1
oAI412_16S rRNA Primer 63f_fwd	TCGTCGGCAGCGTCAGATGTGTATAAGAGACAGGTGGGGTCAGGCCTAACACA	PCR-1
oAI412_mCherry_rev	GTCTCGTGGGCTCGGAGATGTGTATAAGAGACAGTTCTGCATTACGGGGCGTCC	PCR-1
oAI413_LucNrev_fwd	TCGTCGGCAGCGTCAGATGTGTATAAGAGACAGCTTATGCAGTTGCTCTCCGGAT	PCR-1
oAI413_AmpR_rev	GTCTCGTGGGCTCGGAGATGTGTATAAGAGACAGAAGGCTTCACGCAGCTATGTG	PCR-1
oAI413_AmpR_fwd	TCGTCGGCAGCGTCAGATGTGTATAAGAGACAGCACATAGCTGCGTGAAGCCTT	PCR-1
oAI413_16S rRNA 1387_rev	GTCTCGTGGGCTCGGAGATGTGTATAAGAGACAGTAAGACGCCTTGTACACTCCG	PCR-1
oAI413_16S rRNA 1387_fwd	TCGTCGGCAGCGTCAGATGTGTATAAGAGACAGCGGAGTGTACAAGGCGTCTTA	PCR-1
oAI413_16S rRNA 63f_rev	GTCTCGTGGGCTCGGAGATGTGTATAAGAGACAGGGAAGACTTGCATGTGTTAGGC	PCR-1
oAI413_16S rRNA 63f_fwd	TCGTCGGCAGCGTCAGATGTGTATAAGAGACAGGCCTAACACATGCAAGTCTTCC	PCR-1
oAI413_mCherry_rev	GTCTCGTGGGCTCGGAGATGTGTATAAGAGACAGTTCTTCTGCATTACGGGGCAC	PCR-1
oAI414_LucNrev_fwd	TCGTCGGCAGCGTCAGATGTGTATAAGAGACAGACGGGCCTTATGCAGTTGCT	PCR-1
oAI414_AmpR_rev	GTCTCGTGGGCTCGGAGATGTGTATAAGAGACAGACAGCTATGTGGCGCGGTAT	PCR-1
oAI414_AmpR_fwd	TCGTCGGCAGCGTCAGATGTGTATAAGAGACAGATACCGCGCCACATAGCTGT	PCR-1
oAI414_16S rRNA 1387_rev	GTCTCGTGGGCTCGGAGATGTGTATAAGAGACAGTGAAGCCTTGTACACTCCGC	PCR-1
oAI414_16S rRNA 1387_fwd	TCGTCGGCAGCGTCAGATGTGTATAAGAGACAGGCGGAGTGTACAAGGCTTCA	PCR-1
oAI414_16S rRNA Primer 63f_rev	GTCTCGTGGGCTCGGAGATGTGTATAAGAGACAGCATGTGTTAGGCCTGACCGT	PCR-1
oAI414_16S rRNA Primer 63f_fwd	TCGTCGGCAGCGTCAGATGTGTATAAGAGACAGACGGTCAGGCCTAACACATG	PCR-1
oAI414_mCherry_rev	GTCTCGTGGGCTCGGAGATGTGTATAAGAGACAGTCTTCTGCATTACGGGGCTC	PCR-1
Pkd1_TAS R2216W Fwd	TCGTCGGCAGCGTCAGATGTGTATAAGAGACAGTGTAGGGAGCCTGAAGTGGA	PCR-1
Pkd1_TAS R2216W Rev	GTCTCGTGGGCTCGGAGATGTGTATAAGAGACAGCCTGCGTGTCTGACCATACC	PCR-1
N701	CAAGCAGAAGACGGCATACGAGATTCGCCTTAGTCTCGTGGGCTC	PCR-2
N702	CAAGCAGAAGACGGCATACGAGATCTAGTACGGTCTCGTGGGCTC	PCR-2
N703	CAAGCAGAAGACGGCATACGAGATTTCTGCCT GTCTCGTGGGCTC	PCR-2
N704	CAAGCAGAAGACGGCATACGAGATGCTCAGGAGTCTCGTGGGCTC	PCR-2
N705	CAAGCAGAAGACGGCATACGAGATAGGAGTCCGTCTCGTGGGCTC	PCR-2
N706	CAAGCAGAAGACGGCATACGAGATCATGCCTAGTCTCGTGGGCTC	PCR-2
N707	CAAGCAGAAGACGGCATACGAGATGTAGAGAGGTCTCGTGGGCTC	PCR-2
N710	CAAGCAGAAGACGGCATACGAGATCAGCCTCGGTCTCGTGGGCTC	PCR-2
N711	CAAGCAGAAGACGGCATACGAGATTGCCTCTTGTCTCGTGGGCTC	PCR-2
N712	CAAGCAGAAGACGGCATACGAGATTCCTCTACGTCTCGTGGGCTC	PCR-2
N714	CAAGCAGAAGACGGCATACGAGATTCATGAGCGTCTCGTGGGCTC	PCR-2
N715	CAAGCAGAAGACGGCATACGAGATCCTGAGATGTCTCGTGGGCTC	PCR-2
S502	AATGATACGGCGACCACCGAGATCTACACCTCTCTATTCGTCGGCAGCG	PCR-2
S503	AATGATACGGCGACCACCGAGATCTACACTATCCTCTTCGTCGGCAGCG	PCR-2
S505	AATGATACGGCGACCACCGAGATCTACACGTAAGGAGTCGTCGGCAGCG	PCR-2
S506	AATGATACGGCGACCACCGAGATCTACACACTGCATATCGTCGGCAGCG	PCR-2
S507	AATGATACGGCGACCACCGAGATCTACACAAGGAGTATCGTCGGCAGCG	PCR-2

Table S1 (continued)

Primer Name	Primer Sequence (5'-3')	Used for
S508	AATGATACGGCGACCACCGAGATCTACACCTAAGCCTTCGTCGGCAGCG	PCR-2
S510	AATGATACGGCGACCACCGAGATCTACACCGTCTAATTCGTCGGCAGCG	PCR-2
S511	AATGATACGGCGACCACCGAGATCTACACTCTCTCCGTCGTCGGCAGCG	PCR-2
N716	CAAGCAGAAGACGGCATACGAGATTAGCGAGTGTCTCGTGGGCTCGG	PCR-2
N718	CAAGCAGAAGACGGCATACGAGATGTAGCTCCGTCTCGTGGGCTCGG	PCR-2
N719	CAAGCAGAAGACGGCATACGAGATTACTACGCGTCTCGTGGGCTCGG	PCR-2
N720	CAAGCAGAAGACGGCATACGAGATAGGCTCCGGTCTCGTGGGCTCGG	PCR-2
N721	CAAGCAGAAGACGGCATACGAGATGCAGCGTAGTCTCGTGGGCTCGG	PCR-2
N722	CAAGCAGAAGACGGCATACGAGATCTGCGCATGTCTCGTGGGCTCGG	PCR-2
N723	CAAGCAGAAGACGGCATACGAGATGAGCGCTAGTCTCGTGGGCTCGG	PCR-2
N724	CAAGCAGAAGACGGCATACGAGATCGCTCAGTGTCTCGTGGGCTCGG	PCR-2
N726	CAAGCAGAAGACGGCATACGAGATGTCTTAGGGTCTCGTGGGCTCGG	PCR-2
N727	CAAGCAGAAGACGGCATACGAGATACTGATCGGTCTCGTGGGCTCGG	PCR-2
N728	CAAGCAGAAGACGGCATACGAGATTAGCTGCAGTCTCGTGGGCTCGG	PCR-2
N729	CAAGCAGAAGACGGCATACGAGATGACGTCGAGTCTCGTGGGCTCGG	PCR-2
S513	AATGATACGGCGACCACCGAGATCTACACTCGACTAGTCGTCGGCAGCGTC	PCR-2
S515	AATGATACGGCGACCACCGAGATCTACACTTCTAGCTTCGTCGGCAGCGTC	PCR-2
S516	AATGATACGGCGACCACCGAGATCTACACCCTAGAGTTCGTCGGCAGCGTC	PCR-2
S517	AATGATACGGCGACCACCGAGATCTACACGCGTAAGATCGTCGGCAGCGTC	PCR-2
S518	AATGATACGGCGACCACCGAGATCTACACCTATTAAGTCGTCGGCAGCGTC	PCR-2
S520	AATGATACGGCGACCACCGAGATCTACACAAGGCTATTCGTCGGCAGCGTC	PCR-2
S521	AATGATACGGCGACCACCGAGATCTACACGAGCCTTATCGTCGGCAGCGTC	PCR-2
S522	AATGATACGGCGACCACCGAGATCTACACTTATGCGATCGTCGGCAGCGTC	PCR-2
PKD1_c.9320_Exon25_fwd	TCGTGCCCCCAAGCCATGT	LR-PCR
PKD1_c.9320_exon34_rev	AGCCGGACACTCACAGGCT	LR-PCR

Table S2: Guide RNA (gRNA) sequences used for correction of the indicated variants via base editing. Sequences are shown in the 5′→3′ orientation, excluding the PAM sequence.

Table S2

**gRNA Name**	**Mutation**	**gRNA Sequence 5´-3´without PAM**	**Species**	**Gene**
gRNA_1	c.412C>T	CCCATCACGGCAGCCACGCC	human	*PKD1*
gRNA_2	c.1202-9G>A	TTCCCACCAGTCTCCAGCGG	human	*PKD1*
gRNA_3	c.1261C>T	CAGGCAGTAGCAGTGCCCGT	human	*PKD1*
gRNA_4	c.1816C>T	GCAGCCACAGCTGGGCGGGC	human	*PKD1*
gRNA_5	c.1831C>T	GGAGCCAGTACACCTGCAGC	human	*PKD1*
gRNA_6	c.2839C>T	TCCCTACAGTACACGGGCCT	human	*PKD1*
gRNA_7	c.3037G>A	TAACCATGGAGCGGATGAAC	human	*PKD1*
gRNA_8	c.3490G>A	GACTTCAGGGACGGCTCCCC	human	*PKD1*
gRNA_9	c.3955G>A	TCACCAGGAACCCGGCCCAC	human	*PKD1*
gRNA_10	c.3984G>A	TTCGACTGAACCTTCGGGGA	human	*PKD1*
gRNA_11	c.4322A>G	CAAGACAGGAGCCTGGGTCT	human	*PKD1*
gRNA_12	c.4906C>T	CCACCTACAGCCCCTCTATG	human	*PKD1*
gRNA_13	c.5086C>T	CAGCTACACATGGTAGGTGC	human	*PKD1*
gRNA_14	c.5477G>A	CTTTTAGGGGCAGCTGGCCA	human	*PKD1*
gRNA_15	c.6011A>G	CCAGGCGCAGGCGACCCGAG	human	*PKD1*
gRNA_16	c.6031C>T	CTTCTACAGCGAGAAGTACC	human	*PKD1*
gRNA_17	c.6406C>T	CGTGGCCTACGCCACGAAGA	human	*PKD1*
gRNA_18	c.6472C>T	CACCTACAGGGGCAGGACCA	human	*PKD1*
gRNA_19	c.6487C>T	ATCACCGCATCAGCACCTGC	human	*PKD1*
gRNA_20	c.6658C>T	AGCCACGGCAGCACCAGCCG	human	*PKD1*
gRNA_21	c.6743A>G	TCACACTGGCCTGGATGCTC	human	*PKD1*
gRNA_22	c.6916-9G>A	CTCAGCCTGCAGAGGGAGGC	human	*PKD1*
gRNA_23	c.7244T>C	TGCACGCACGTTCAGCAACA	human	*PKD1*
gRNA_24	c.7271C>T	GCCCATGGATGTGGTGGTCT	human	*PKD1*
gRNA_25	c.7915C>T	GGGCTCAGTGCTGCCGCTCG	human	*PKD1*
gRNA_26	c.7949T>C	GAGACTCCGGTGTCCCTGAG	human	*PKD1*
gRNA_27	c.7984C>T	CTGCTAGATGTCATCCACAG	human	*PKD1*
gRNA_28	c.8311G>A	CAACAAGGAGCCCCTGACGC	human	*PKD1*
gRNA_29	c.8381T>C	AGCCTGCCGTGCTATGGCGG	human	*PKD1*
gRNA_30	c.8558T>C	ATGGCATCCCAGACACAGGC	human	*PKD1*
gRNA_31	c.8593C>T	CAGCCACTCGATGGGGATCT	human	*PKD1*
gRNA_32	c.8929A>G	AAGGCGTAGGGCCGGTGGTC	human	*PKD1*
gRNA_33	c.9335G>A	CTTTCTATGGGCAGCGGGGC	human	*PKD1*
gRNA_34	c.9340C>T	CCGCTACCCACAGAAAGGGA	human	*PKD1*
gRNA_35	c.9547C>T	CCACACTCAGATCTTCCACA	human	*PKD1*
gRNA_36	c.9851G>A	CCTGCTACGTTCTCCTCATC	human	*PKD1*
gRNA_37	c.10290G>A	CCATTGTAGGTAGCAATCTG	human	*PKD1*
gRNA_38	c.10945C>T	CCGTGGGATGGCCGTACGCG	human	*PKD1*
gRNA_39	c.10951G>A	CCACAGCTTTGCACTCTTCC	human	*PKD1*
gRNA_40	c.11156+3A>G	GCCCGCACCGCGTGATGGCC	human	*PKD1*
gRNA_41	c.11384G>A	GACGTAGGCCTATTCAGCGC	human	*PKD1*
gRNA_42	c.12010C>T	TGCTAGGCAGCCTGCGGACG	human	*PKD1*
gRNA_43	c.12061C>T	CAGAGCTCAGCATAATGTCT	human	*PKD1*
gRNA_44	c.12124C>T	CAGCTAGGCGTAGGCTACCC	human	*PKD1*
gRNA_45	c.6646C>T_1	AGCCATGGCACCACCAGCTG	mouse	*Pkd1*
gRNA_46	c.6646C>T_2	CAGCCATGGCACCACCAGCT	mouse	*Pkd1*
gRNA_47	c.6646C>T_3	CCAGCCATGGCACCACCAGC	mouse	*Pkd1*
gRNA_48	c.6646C>T_4	GCCATGGCACCACCAGCTGG	mouse	*Pkd1*
