# Supplementary material for: In vivo base editing reduces liver cysts in autosomal dominant polycystic kidney disease
Source: Mol Ther. 2025 Aug 20;33(11):5373–82. doi: 10.1016/j.ymthe.2025.08.026 (PMC12628146; doi:10.1016/j.ymthe.2025.08.026)
Supplement: Document S2. Article plus supplemental information [file mmc2.pdf]

Molecular Therapy
Brief report

In vivo base editing reduces liver cysts in autosomal dominant polycystic kidney disease

Antonia Ibel,[1,2,8] Rishi Bhardwaj,[3,8] Duygu Elif Yilmaz,[1] Shuhan Kong,[1,2] Sarah Wendlinger,[1,2] Carlos Cordero,[1,2] Dimitra Papaioannou,[1,2] Maria Papazian,[1] Ria Schönauer,[1] Qiyao Meng,[1,2] Kai-Uwe Eckardt,[1] Fatima Hassan,[3] Isabel Volpe,[3] Verena Klämbt,[4,5] Jan Halbritter,[1] Sorin Fedeles,[3,7] Matteus Krappitz,[1,5,6,7] and Michael M. Kaminski[1,2,5,7]

[1]Charité – Universitätsmedizin Berlin, Corporate Member of Freie Universität Berlin and Humboldt-Universität zu Berlin, Department of Nephrology and Medical Intensive Care, Charitéplatz 1, 10117 Berlin, Germany; [2]BIMSB, Max Delbrück Center for Molecular Medicine in the Helmholtz Association, 10115 Berlin, Germany; [3]Department of Internal Medicine, Section of Nephrology, Yale School of Medicine, New Haven, CT 06520-8029, USA; [4]Charité – Universitätsmedizin Berlin, Corporate Member of Freie Universität Berlin and Humboldt-Universität zu Berlin, Department of Pediatric Gastroenterology, Nephrology, and Metabolic Diseases, Augustenburger Platz 1, 13353 Berlin, Germany; [5]Berlin Institute of Health, BIH Charité Clinician Scientist Program, 10178 Berlin, Germany; [6]Division of Renal Diseases and Hypertension, School of Medicine, CU Anschutz, University of Colorado, Aurora, CO 80045, USA

Autosomal dominant polycystic kidney disease (ADPKD) is the most prevalent genetic kidney disorder, affecting over 10 million individuals worldwide. Cystic expansion typically progresses to kidney failure and also involves the liver with limited treatment options. Pathogenic variants in *PKD1* or *PKD2* account for 85%–90% of cases. Genetic re-expression of *Pkd1* or *Pkd2* has been shown to partially reverse key characteristics of the disease phenotype in mice. Despite advancements in understanding the genetic basis, it remains unclear whether correcting pathogenic variants can effectively prevent, modify, or reverse the disease. Additionally, the feasibility of genome editing as a treatment remains largely unexplored. In this study, we employed CRISPR base editing to correct representative pathogenic *PKD1* variants selected from a patient cohort, achieving precise and efficient editing *in vitro*. Correction of a murine missense variant (c.6646C>T (R2216W)) in primary renal epithelial cells increased polycystin-1 expression and reduced the endoplasmic reticulum stress marker sXBP1. *In vivo*, base editor delivery to the c.6646C>T (R2216W) knockin mouse enabled correction of the pathogenic variant, resulting in a significant reduction in liver cysts. These findings provide the first evidence that genome editing may ameliorate key features of ADPKD, opening promising therapeutic perspectives for affected patients and their families.

INTRODUCTION

Autosomal dominant polycystic kidney disease (ADPKD) is the most prevalent genetic cause of chronic kidney disease, with a prevalence between 1 in 400 and 1 in 1,000 individuals.[1,2] ADPKD affects over 10 million people globally and thus represents a significant health burden. It is a multisystemic disorder characterized by polycystic kidney and liver disease (PLD) along with additional extrarenal manifestations such as intracranial arterial aneurysms. Renal cysts originate from multiple tubular segments of the nephron, gradually expanding over the lifetime. This growth compresses the surrounding renal tubules, leading to cystic kidney enlargement, inflammation, fibrosis, and progression to kidney failure, typically occurring between the ages of 40 and 70 years.[1,2] Common complications of liver or kidney cysts include compression of the neighboring intraperitoneal (i.p.) and intrathoracic organs, infections, and pain. Tolvaptan, a vasopressin V2 receptor antagonist, is the only approved therapy to slow disease progression, albeit with limited efficacy and severe side effects. Importantly, Tolvaptan does not affect liver cysts, leaving patients with progressive liver cysts without any pharmacological treatment option. Because at least 10% of ADPKD patients suffer from symptomatic PLD, there is an urgent medical need for developing treatment options. PLD is the most frequent extrarenal manifestation of ADPKD and may lead to abdominal fullness, pain, lack of appetite, and sarcopenia despite preserved liver function. These symptoms result from the mass effect of severely enlarged cystic livers compressing adjacent gastrointestinal organs.[3,4]

Received 22 March 2025; accepted 18 August 2025;
https://doi.org/10.1016/j.ymthe.2025.08.026.

[7]These authors contributed equally

[8]These authors contributed equally

Correspondence: Sorin Fedeles, Department of Internal Medicine, Section of Nephrology, Yale School of Medicine, New Haven, CT 06520-8029, USA.
E-mail: sorin.fedeles@yale.edu
Correspondence: Matteus Krappitz, Division of Renal Diseases and Hypertension, School of Medicine, CU Anschutz, University of Colorado, Aurora, CO 80045, USA.
E-mail: matteus.krappitz@cuanschutz.edu
Correspondence: Michael M. Kaminski, Charité – Universitätsmedizin Berlin, Corporate Member of Freie Universität Berlin and Humboldt-Universität zu Berlin, Department of Nephrology and Medical Intensive Care, Charitéplatz 1, 10117 Berlin, Germany.
E-mail: michael.kaminski@charite.de

© 2025 The Authors. Published by Elsevier Inc. on behalf of The American Society of Gene and Cell Therapy.
This is an open access article under the CC BY license (http://creativecommons.org/licenses/by/4.0/).

Around 85%–90% of ADPKD cases are due to variants in *PKD1* (75%) or *PKD2* (10%–15%). Patients with *PKD1* variants generally exhibit an earlier onset and more severe disease progression. *PKD1* and *PKD2* encode polycystin-1 (PC1) and PC2, respectively, key components of a calcium-permeable ion channel in renal tubular cells that are crucial for intracellular signaling in primary cilia. Importantly, the 5′ two-thirds of the human *PKD1* gene (exons 1–33) lie within a segmentally duplicated region on chromosome 16p13 that has been copied into 6 highly homologous pseudogenes (PKD1P1–PKD1P6), which share ∼97.7% sequence identity with the functional gene and therefore complicate accurate variant detection in human studies.[5,6] Additionally, pathogenic variants in genes such as *IFT140*, *GANAB*, *ALG5*, *ALG8*, *ALG9*, or *DNAJB11* account for less than 1% of ADPKD-like phenotypes. ADPKD patients typically carry a heterozygous *PKD1* or *PKD2* germline mutation, and cyst formation and disease progression often require a "second hit," which may involve somatic inactivation of the wild-type *PKD1* or *PKD2* allele, variants in other ADPKD-related genes, environmental factors, or unidentified genetic modifiers. Additionally, it has been suggested that cystogenesis in ADPKD is influenced by gene dosage thresholds, with disease severity correlating to functional levels of polycystin proteins.[7,8] Consequently, loss-of-function variants are associated with more severe phenotypes, whereas milder, late-onset forms of the disease are linked to hypomorphic missense mutations that partially preserve polycystin function.[1,9] Studies have demonstrated that reduced levels of PC1 in animal models are sufficient to induce renal cyst formation. Conversely, genetic reactivation of *Pkd1* or *Pkd2* in murine ADPKD mouse models results in a partial reversal of the phenotype.[10] Additionally, the genetic deletion or pharmaceutical inhibition of the miR-17 motif within the 3′ UTR of the *PKD1* or *PKD2* genes, which increases PC1 and PC2 levels, attenuated renal cyst growth in an experimental *Pkd1*-mutant mouse model, even after disease onset.[11] While re-expression of *Pkd1* or *Pkd2* in mouse models can reverse certain aspects of ADPKD, even in advanced stages,[10–12] it is unknown whether direct correction of pathogenic *PKD1* variants through genome editing may similarly restore *PKD1* function. Base editors are CRISPR-Cas-based genome editing tools that enable the direct conversion of single bases without the need of double-strand DNA (dsDNA) breaks, DNA templates, or homology-directed repair.[13,14] They consist of a catalytic disabled Cas enzyme fused to a deaminase, enabling a C>T,[13] A>G,[14] A>Y,[15] and C>G[16] edit. Recent preclinical studies have highlighted their potential and safety profile for *in vivo* genome editing in various genetic diseases.[17,18] However, no genome editing approach has been applied to prevent, halt, or reverse ADPKD.

Here, we explore base editing as a potential treatment of ADPKD by focusing on its most important extrarenal complication: polycystic liver disease.

RESULTS

Correction of pathogenic *PKD1* variants selected from an ADPKD patient cohort using adenine and cytosine base editing

Point mutations represent the largest category of human pathogenic variants, accounting for 58% of all pathogenic genetic changes.[19] Common base editors, such as adenine base editors (ABEs) and cytosine base editors (CBEs), could theoretically correct up to 61% of these mutations. To explore the applicability of base editing for pathogenic *PKD1* variants, we screened our local ADPKD cohort (Figure 1A) and identified 39 representative variants distributed across exons 4–43. These included both missense and nonsense variants, which were potentially targetable with CBEs or ABEs (Figure 1B). The variants were introduced into HEK293T cells using a transposase system, which stably integrated the pathogenic variant along with 150 bp of flanking *PKD1* genomic sequence (Figure 1A). We tested different combinations of BEs and single-guide RNAs (sgRNAs) with corresponding protospacer-adjacent motifs (PAMs) for optimal editing (Figure 1C). While editing of only five variants showed correction efficiencies below 10%, editing of most variants achieved on-target efficiencies between 30% and 60%, comparable to the editing efficiency observed at a commonly used, highly efficient control site in the β2-microglobulin (*B2M*) gene (Figure 1C).[20] Notably, as part of our screening approach, we did not enrich for successfully transfected cells, likely leading to an underestimation of editing efficiency. Furthermore, to confirm the applicability of our findings to physiological conditions, we isolated human urine-derived renal epithelial cells (hUREC) from a patient carrying the *PKD1* c.9340C>T variant and established a sequencing-based readout that excludes *PKD1* pseudogenes (Figure 1D). Thereby, we could reliably detect the c.9340C>T mutation in these cells and observed efficient correction of the mutant allele upon base editing (Figure 1E). These findings indicate that BEs can be broadly applied to correct pathogenic *PKD1* variants in ADPKD, including those affecting the most functionally relevant domains (e.g., REJ, PLAT), underscoring their clinical potential.

To optimize on-target editing and reduce bystander editing, we selected the hypomorphic c.6658C>T (R2220W) pathogenic missense variant from our screen, for which a fully characterized knockin mouse model is available, mimicking key characteristics of human ADPKD.[21] In engineered HEK293T cells, we tested three different BEs (ABEmax, ABE8.20m, and ABE8e) with varying editing windows, in combination with suitable sgRNAs, to assess efficiency and precision for correction of the human c.6658C>T (R2220W) (Figure 2A; Table S2). The observed editing efficiencies for the intended A>G conversion ranged from 10% to 65%, with ABE8e demonstrating the highest efficiency. We then assessed editing outcomes for potential unwanted bystander mutations and identified A>G edits at position A10 and A13 of the protospacer sequence (numbered from 1 to 20 in the 5′–3′ direction, with the PAM as positions 21–23; Figure 2A). The A10>G edit leads to a leucine to proline substitution at amino acid position p.2218, while the A13>G edit results in a valine to alanine substitution at amino acid position p.2217. This bystander edit was predominantly observed with ABE8e or ABE8.20m, while ABEmax achieved 20.9% ± 7.9% on-target editing efficiency without any bystander mutations and consequently was selected for further experiments. These findings demonstrate that the correction of c.6658C>T (R2220W) can be systematically optimized, ultimately yielding efficient and precise base editing using ABEmax.

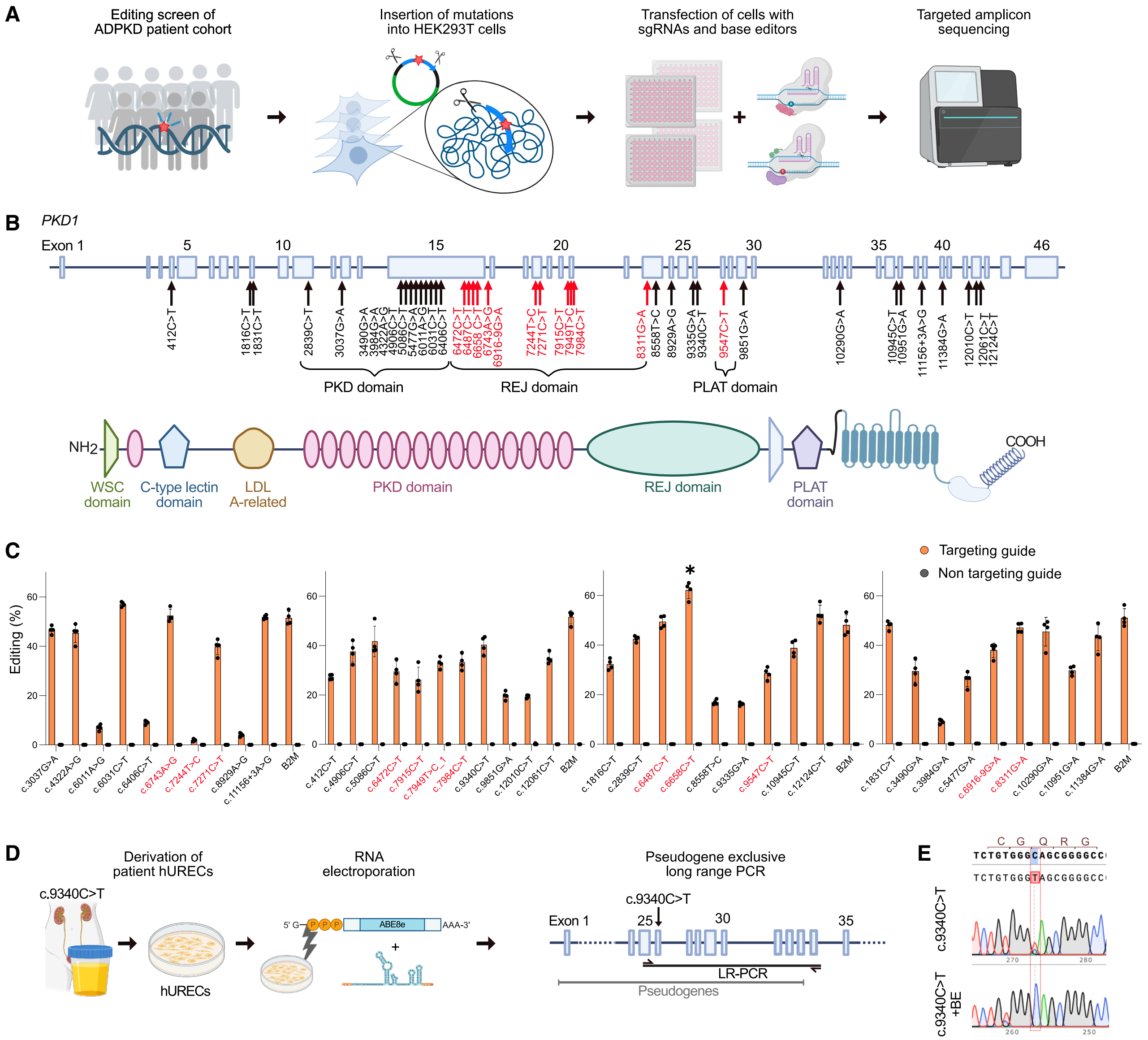

Figure 1. Base editing screen for correction of *PKD1* variants

(A) Schematic indicating the experimental workflow. From 185 patients with typical ADPKD, 104 were selected with (likely) pathogenic single-nucleotide variants, from which 39 variants correctable by adenine or cytosine base editors (BEs) were introduced in HEK293T cells along with 150 bp of flanking endogenous sequence using a transposase system. Cells were then transfected with BEs and sgRNAs, followed by targeted amplicon sequencing. (B) Genomic structure and protein domain structure of *PKD1*. Arrows indicate genomic positions of identified pathogenic variants from our ADPKD cohort; red indicates location in functionally important domains (e.g., REJ, PLAT). (C) Editing efficiency across all different *PKD1* variants identified in our ADPKD cohort, tested in four different engineered HEK293T cell lines. Separate graphs indicate separate cell lines. β2-Microglobulin (*B2M*) served as a positive control. Asterisk highlights human c.6658C>T variant (R2220W, corresponding to murine R2216W) showing the highest editing efficiency. $n = 4$ biological replicates. Bar graphs indicate means ± SDs. (D) Schematic of the experimental workflow for base editing in hURECs. Long-range PCR (LR-PCR) spanning exons 26–34 was performed to exclude *PKD1* pseudogenes from analysis. (E) Sanger sequencing of *PKD1* c.9340C>T mutant cells compared to BE-treated cells.

Functional characterization of *in vitro* base editing outcomes for correction of the murine *Pkd1* c.6646C>T (R2216W) variant

Next, we aimed to correct the corresponding mouse variant c.6646C>T (R2216W) in primary renal epithelial cells (RTECs) derived from the $Pkd1^{R2216W/-}$ knockin mouse model (Figure 2B). In these mice, the pathogenic variant is carried on one allele, while the other allele is deactivated through Cre-mediated excision of exons 2–4 upstream of the variant's position, resulting in heterozygosity for the variant (Figure 2C). ABEmax and sgRNA_45 yielded a mean cytosine recovery of 68.7% ± 15.4%, compared to 33.7% ± 1.5% in non-edited control cells, as determined by next-generation targeted amplicon sequencing (TAS) (Figure 2B; Table S2). We

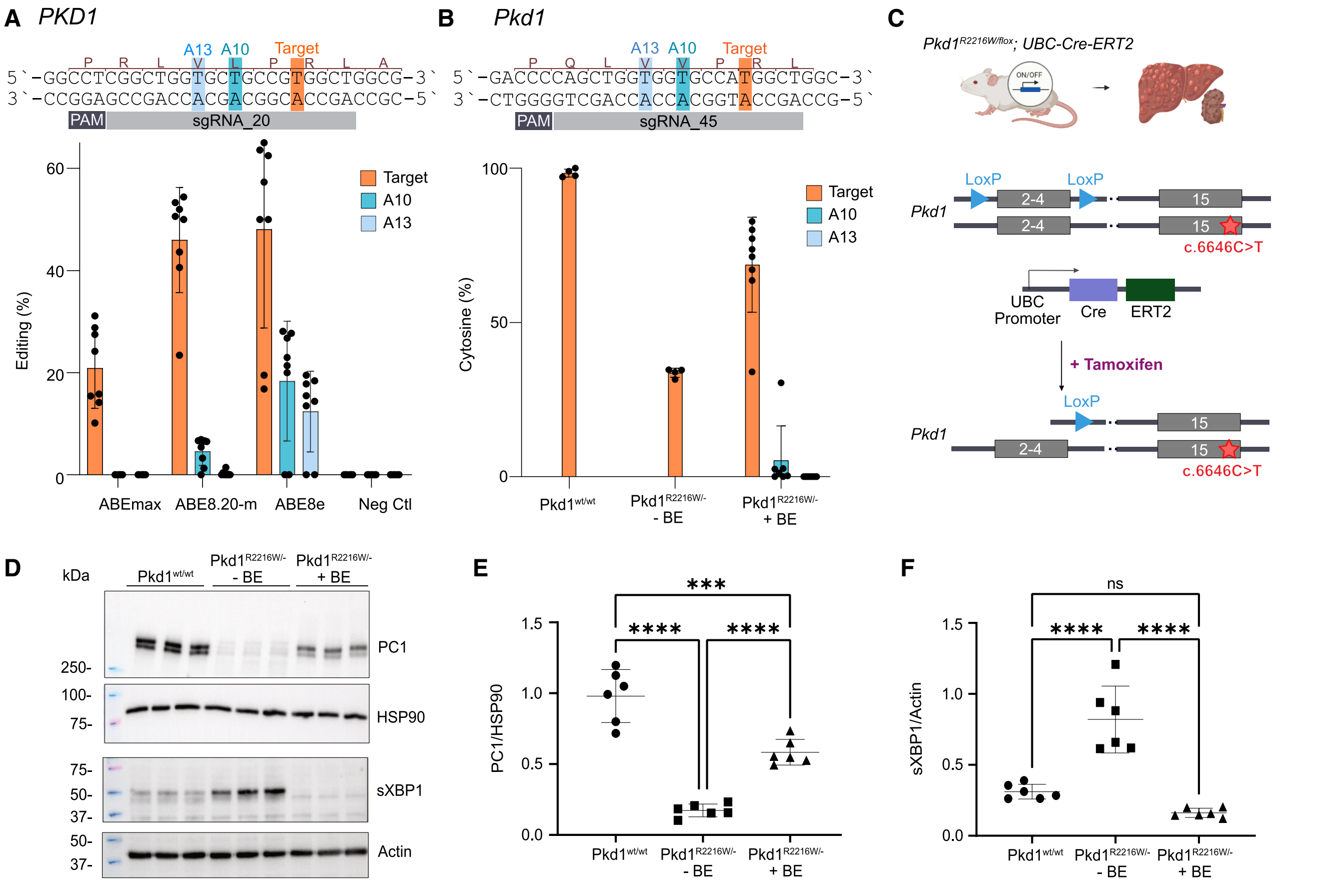

Figure 2. Correction and functional characterization of the RW missense variant *in vitro*

(A) Schematic illustrating the targeting strategy; editing efficiency is shown for indicated BEs and sites in human *PKD1* c.6658C>T (R2220W) in engineered HEK293T cells. Orange indicates the intended on-target editing, and blue indicates unintended bystander editing. Pooled data from 2 experiments with $n = 4$ independent replicates. Data are the means ± SDs. (B) Schematic illustrating the targeting strategy; editing efficiency is shown for indicated BEs and sites in corresponding mouse *Pkd1* c.6646C>T (R2216W) in RTEC cells. Data points represent different (polyclonal) cell populations enriched for successful transfection by FACS from independent replicates. Data are the means ± SDs. (C) Schematic of the knockin mouse model with the pathogenic *Pkd1* c.6646C>T (R2216W) on one allele and deactivation of the other allele through Cre-mediated excision of exons 2–4. *UBC* driven Cre expression upon tamoxifen administration leads to cystic liver disease and mild cystic kidney disease. (D) Western blots showing levels of the indicated proteins in BE-treated cells and controls. sXBP1 served as an ER stress marker. Shown are representative immunoblots for PC1 (NTF fragment, 450 kDa) and spliced XBP1 (sXBP1, 56 kDa); HSP90 or β-actin served as loading controls (90 and 42 kDa, respectively) and are shown below the respective immunoblots. (E and F) Graphs showing densitometric quantification of PC1 and sXBP1 relative to the corresponding loading controls; $n = 3$ biological replicates with two technical replicates. Data are the means ± SDs; ***$p < 0.001$; ****$p < 0.0001$; ns, not significant.

observed no bystander editing at A13 (A13>G) and minimal bystander editing at A10 (A10>G) of 5.3% ± 11.2%, resulting in a valine-to-alanine substitution at amino acid position p.2214. Following base editing, we next confirmed the restoration of PC1 expression by immunoblotting, which showed recovery of PC1 protein levels (Figure 2D). Deglycosylation analysis of PC1 in both BE-treated and control cells revealed similar fractions of mature (Endo H-resistant) and immature (Endo H-sensitive) glycoforms (Figure S2B). The mature Endo H-resistant glycoform accounted for more than 90% of total PC1, indicating that the mature isoform is the predominant form also in the edited cells. In addition to rescuing PC1 expression, base editing significantly reduced the expression of the endoplasmic reticulum (ER) stress marker sXBP1, upregulated in unedited controls.[22,23] Densitometric analysis indicated a marked improvement in PC1 levels (Figure 2E) and a significant reduction in sXBP1 (Figure 2F), demonstrating that base editing not only restored the protein expression but also mitigated the associated cellular stress response induced by the pathogenic variant.

In vivo correction of *Pkd1* c.6646C>T (R2216W)

Next, we aimed to evaluate whether base editing can correct the pathogenic *Pkd1* c.6646C>T (R2216W) variant *in vivo* and potentially halt or reverse the phenotypic manifestations of ADPKD. Besides the primary kidney manifestation, cystic liver disease presents a major therapeutic challenge in many cases of ADPKD. Therefore, we selected an ADPKD model that develops both kidney and liver

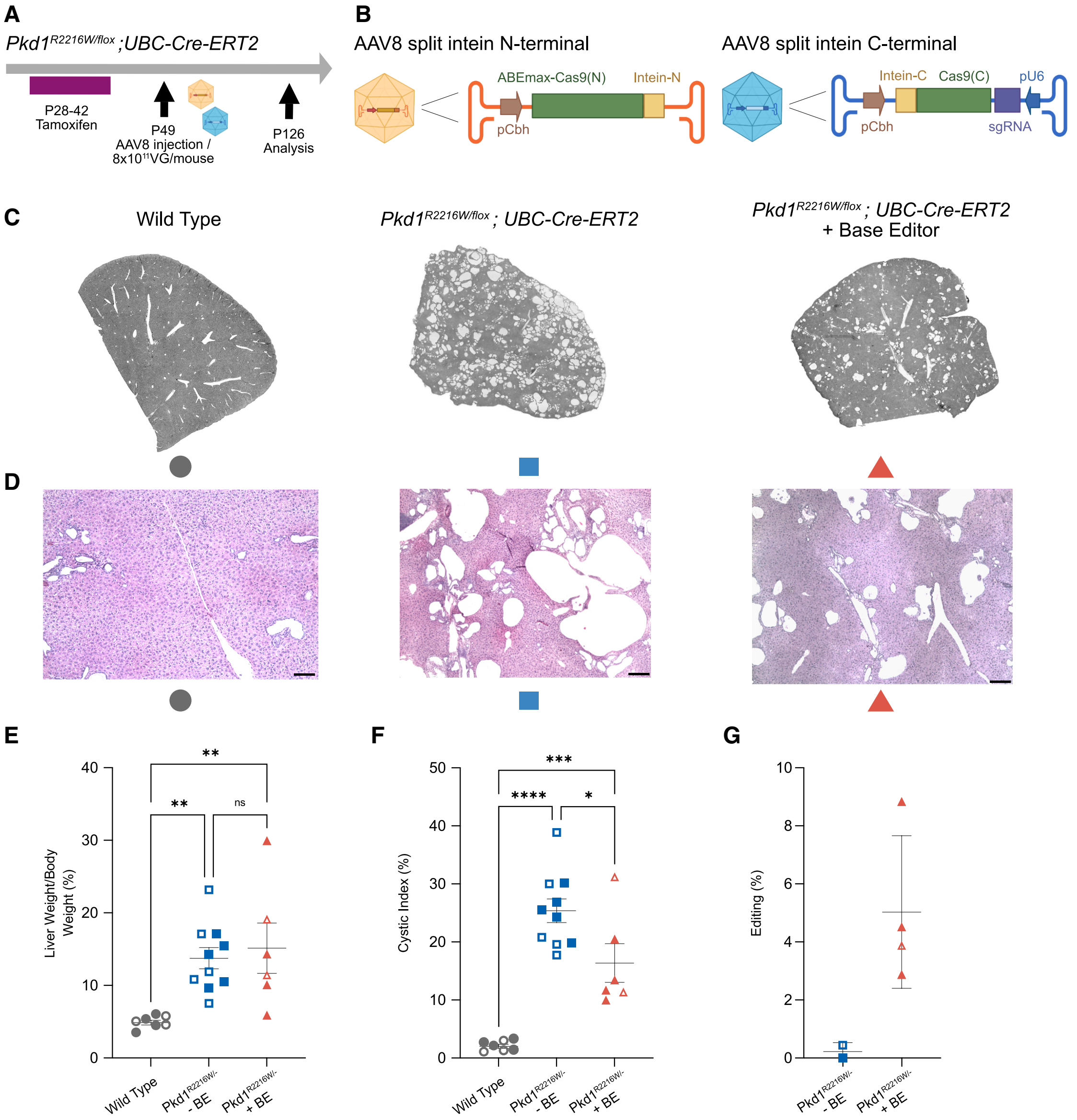

Figure 3. *In vivo* base editing reduces liver cysts in the RW knockin model

(A) Schematic illustrating the experimental workflow of tamoxifen-mediated Cre expression; AAV8 delivered base editing system and analysis at the indicated postnatal (P) days. (B) Schematic illustrating split-intein-based AAV8 delivery of ABEmax. (C) Microscopic overview images of liver tissue from the indicated conditions. (D) Hematoxylin and eosin stain of the indicated conditions. Scale bar = 200 μm. (E and F) Quantification of liver-to-body weight ratios (E) and liver cystic indices (F) between wild-type mice (n = 7), untreated RW knockin mice (n = 10), and BE-treated RW knockin mice (n = 6). (G) Quantification of editing efficiency in gDNA isolated from the liver of the untreated RW knockin mice (n = 2) and BE-treated RW knockin mice (n = 4). (E–G) Data points represent independent biological replicates with means ± SDs; *$p < 0.05$; **$p < 0.01$; ***$p < 0.001$; **** $p < 0.0001$; ns, not significant. Filled data points indicate male mice; open symbols indicate female mice.

cysts. Importantly, this model enables the evaluation of base editing for ADPKD independently of kidney delivery constraints, as liver delivery is well established. *Pkd1*$^{R2216W/fl}$;*UBC-Cre-ERT2* mice were treated with tamoxifen for 14 days starting at postnatal day 28 (P28), leading to Cre-mediated excision of the floxed allele and resulting in a marked cystic liver phenotype and a mild cystic kidney phenotype (Figure 3A). At P49, these mice were injected i.p. with adeno-associated viruses (AAVs) at 8×10^{11} viral genomes (VG) per mouse carrying base editing components, followed by analysis at P126. We selected a dual AAV8 approach delivering a split-intein ABEmax together with sgRNA_45 (Figure 3B; Table S2).[24] Histological analysis of untreated mice revealed extensively cystic liver tissue affecting all segments and disrupting tissue architecture, whereas BE-treated mice exhibited only mild phenotypic changes

(Figures 3C, 3D, and S3A). While liver-to-body weight ratios showed no significant differences between groups (Figure 3E), cystic indices were significantly lower in ABEmax-treated mice compared to untreated controls (Figure 3F), highlighting the potential of base editing to reduce cystic burden in ADPKD. Due to the mild cystic kidney phenotype in control mice and the liver tropism of AAV8 resulting in high hepatic transduction efficiency but low renal transduction efficiency (Figures S2C and S2D), kidney-to-body weight ratios and kidney cystic indices did not differ between the groups (Figures S3B–S3D). Next, we explored which editing efficiencies correlated with the observed phenotypic differences. In liver tissue, we observed a mean on-target editing of 5.0% ± 2.6%, compared to 0.5% ± 0.1% in non-edited control mice (Figure 3G), indicating that an overall low editing efficiency is sufficient to modify the cystic liver phenotype. Importantly, no unwanted bystander mutations were detected at A10 or A13 within the editing window. Finally, we examined sgRNA-dependent off-target editing. *In silico* prediction using Cas-OFFinder[25] identified two sites for the human c.6658C>T (R2220W) variant with one and two mismatches, all identified to be located in *PKD1* pseudogenes, and 31 sites with three mismatches, all without RNA or DNA bulges. For the mouse variant c.6646C>T (R2216W), one site with one mismatch was found, and no two-mismatch sites and 21 three-mismatch sites were identified. TAS of the top six predicted off-target sites showed no editing in the liver, indicating precise base editing (Figures S1A–S1C).

DISCUSSION

This study presents the first *in vivo* evidence of using base editing to correct pathogenic PC1 variants in ADPKD, providing promising results for the potential reversal of cystic liver disease. Our findings demonstrate that base editing can be applied broadly to human *PKD1* mutations, with correction efficiencies ranging from 30% to 60% for most variants in HEK293T cells. The restoration of PC1 expression in $Pkd1^{R2216W/-}$ cells after base editing provides further validation of the potential of ABE to restore the normal function of proteins compromised by genetic mutations. The reduction of the ER stress marker sXBP1 further indicates that base editing can alleviate cellular stress associated with pathogenic *PKD1* variants, an important factor in disease progression. These results suggest that ABE not only corrects the underlying genetic defect but also positively impacts disease-related molecular pathways. Our *in vivo* experiments, conducted in $Pkd1^{R2216W/fl}$;*UBC-Cre-ERT2* mice, demonstrated a significant reduction in liver cystic burden following a single administration of AAV8-ABE, providing strong initial evidence for the therapeutic potential of base editing in ADPKD. Notably, the editing efficiency required to improve the cystic liver phenotype *in vivo* was comparatively low, underscoring the overall feasibility of this approach. These data support that early intervention targeting a limited fraction of disease-initiating cells may produce phenotypic improvement in ADPKD. While our gDNA editing measurement represents a single time point and may underestimate true editing dynamics, similar partial genomic corrections in other diseases have been sufficient to alter disease progression, underscoring a promising therapeutic window for genome editing in ADPKD. Although the mouse model and the delivery vehicles used here were not designed to investigate the kidney phenotype, we observed a trend toward a reduced kidney cyst burden and low editing in some BE-treated kidneys (Figure S3E). Along with recently reported AAV serotypes and the potential to further increase the AAV dose,[26,27] this may support the feasibility of targeting renal disease in ADPKD, which represents the major cause of morbidity in affected patients. Our data suggest that base editing can be used to correct pathogenic mutations in a complex, multiorgan genetic disease. Unlike conventional treatments such as tolvaptan, which slow disease progression but do not address the underlying genetic cause, BEs offer the possibility of potentially reversing disease phenotypes by maintaining or restoring normal protein function at the genetic level. The significant reductions in liver cyst burden observed in our study further highlight the potential of base editing to treat extrarenal complications of the disease, which significantly impact morbidity and mortality but remain unaddressed by tolvaptan, the only licensed medical treatment to date. While the present study demonstrates the feasibility and therapeutic potential of BEs in ADPKD, several important questions remain. Notably, we observed substantial variability in the liver-to-body weight ratio, which may reflect differences in disease penetrance associated with variability in Cre activation or genetic backgrounds in our model. Systemic physiological variation affecting body weight independently of liver pathology may further contribute to this variability. In addition, optimization of delivery methods, particularly to enhance editing efficiency in kidney tubular epithelial cells, will be crucial. Further work is needed to assess long-term outcomes, genomic stability, and potential off-target effects associated with AAV8-ABE delivery. While the overall low *in vivo* editing efficiency was sufficient to alter the hepatic phenotype, improvements in BEs are likely to enhance editing efficiency and phenotypic rescue. Notably, we selected ABEmax due to its lowest degree of bystander editing; however, more active BEs, such as ABE8.20m and ABE8e, are likely to increase editing efficiency, as suggested by our *in vitro* data (Figure 2A). While we used AAV at a dose of 8×10^{11} VG per mouse (approximately 4×10^{13} VG/kg) to avoid liver toxicity, recent reports suggest that higher doses could further enhance editing efficiency.[28] Finally, recent studies have demonstrated the potential of lipid nanoparticles for highly efficient delivery of BEs to the liver, offering a scalable and increasingly safe method for therapeutic PLD applications.[29,30]

In conclusion, this study provides initial evidence that adenine base editing is a promising therapeutic strategy for ADPKD. By successfully correcting *PKD1* mutations *in vitro* and *in vivo*, we have demonstrated the potential of base editing to address the genetic root of the disease and reverse cystic phenotypes in the liver. Future work will focus on optimizing delivery to the kidney, increasing editing efficiency, and exploring the full therapeutic potential of ABEs in preventing and treating ADPKD.

MATERIALS AND METHODS

Cloning strategy and plasmids

sgRNA sequences were synthesized as dsDNA fragments (Eurofins Genomics). The duplexed oligos of the corresponding

spacer sequence were annealed and ligated into the BsmBI-digested pU6-pegRNA-GG-acceptor plasmid provided by David Liu (Addgene plasmid no. 132777). pCMV_BE4max, pCMV_ABEmax, and ABE8e were a gift from David Liu (Addgene plasmid nos. 112093, 112095, and 138489). ABE8.20-m was a gift from Nicole Gaudelli (Addgene plasmid #no. 136300). pCMV-T7-ABE8e-nSpG-P2AEGFP (KAC984), pCAG-CBE4max-SpG-P2A-EGFP (RTW4552), and pCMV-T7-ABE8enSpRY-P2A-EGFP (KAC1069) were gifts from Benjamin Kleinstiver (Addgene plasmid nos. 185911, 139998, and 185912).[20,31–35]

Cell culture, transfection, and DNA isolation

HEK293T cell lines were maintained in DMEM (Thermo Fisher Scientific) supplemented with 10% (v/v) fetal bovine serum (Sigma F7524) and 1% penicillin/streptomycin (P/S) at 37°C with 5% CO_2. HEK293T cell lines were seeded at low passages with a density of 5×10^5 cells/300 μL in antibiotic-free medium in a 48-well cell culture microplate (Falcon CLS351172) and grown for 24 h at 37°C with 5% CO_2. Transfection with sgRNA and BE plasmids was performed using the TransIT-X2 Dynamic Delivery System (Mirus Bio, catalog no. MIR 6004) following the manufacturer's protocol in a 3:1 BE:sgRNA ratio by weight (Table S2). We transfected 260 ng total plasmid per well, and 72 h after transfection, genomic DNA (gDNA) isolation was performed. For HEK293T cell line gDNA isolation, cells were washed once with PBS (Dulbecco's PBS, Gibco, catalog no. 14190250), trypsinized, spun down at 250 × *g* for 5 min. Pellets were resuspended in 100 μL lysis buffer and incubated for 16 h at 55°C on a heat block at 300 rpm. gDNA was isolated and purified using MagBinding Beads (Zymo Research, catalog no. D4100-2-24) at a 1× ratio, with three washing steps using 80% ethanol and eluted in EB buffer. MagBinding Beads were prepared according to the manufacturer's protocol. For quantification of gDNA, the Quant-iT dsDNA high-sensitivity (HS) assay (Invitrogen, catalog no. Q33232) was used. RTECs used in this study have been described previously and were isolated from $Pkd1^{R2216W/-}$ mice as reported before.[23,36,37] In brief, kidneys were harvested and subjected to enzymatic digestion with collagenase type II to dissociate the tissue. The nephron segments were then microdissected in PBS under a stereomicroscope, cultured, and after reaching confluence, the cells were subjected to limiting dilution to isolate and expand a clone derived from a single cell. RTECs were maintained in DMEM/F12 (Thermo Fisher Scientific) supplemented with 10% (v/v) fetal bovine serum (Sigma, catalog no. F7524) and 1% P/S at 37°C with 5% CO_2. RTECs were seeded at low passages with a density of 4×10^5 cells/2 mL in antibiotic-free medium in a 6-well cell culture microplate (Falcon, catalog no. CLS351172) and grown for 24 h at 37°C with 5% CO_2. Transfection with sgRNA and BE plasmids was performed using the TransIT-X2 Dynamic Delivery System (Mirus Bio, catalog no. MIR 6004) following the manufacturer's protocol in a 3:1 BE: sgRNA ratio by weight (Table S2). 2.5 μg total plasmid DNA was transfected per well. At 72 h after transfection cells were single-cell sorted for GFP expression and colonies of GFP^+ cells were expanded and sequenced. RTEC gDNA isolation was performed as mentioned above.

TAS

Next-generation TAS was performed to determine the efficiency of genome modification at the target sites using a two-step PCR-based library construction method adapted from the Illumina Nextera XT DNA library preparation. The target loci were amplified from 100 ng gDNA using the Q5 Hot Start High Fidelity 2× MM (New England Biolabs) and PCR-1 primers (Table S1). Primers for the engineered HEK293T cell lines were used according to the respective construct or designed with PrimerBlast by NCBI with overhangs allowing for the subsequent indexing PCR. The PCR products were purified using MagBinding Beads (Zymo Research, catalog no. D4100-2-24) at a 0.8× ratio and quantified using the Quant-iT dsDNA HS assay. 20 ng of purified PCR-1 products was used as template for the indexing PCR (PCR-2) to add barcodes and Illumina adapter sequences using Q5 and primers (Table S1). PCR products were again purified using MagBinding Beads at a 0.7× ratio, quantified, and pooled equimolar. Pooled libraries were checked with the D1000 ScreenTape system (Agilent), spiked with 30%–60% PhiX (Illumina), depending on the library complexity, and subsequently denatured. The final library was loaded on a MiniSeq sequencer at 1.5 pM and sequenced using a MiniSeq Mid Output Kit (300 cycles) (Illumina). On-target genome-editing efficiencies and bystander edits were determined from sequencing data using CRISPResso2.[38]

Generation of HEK293T reporter cell line

HEK293T cell lines were engineered to carry pathogenic *PKD1* variants by using the Sleeping Beauty transposase system.[39] The variants, flanked by 150 bp of surrounding genomic sequence, including specific primer sequences on the 5′ and 3′ ends (Table S1) for targeted sequencing, were synthesized as four dsDNA fragments (IDT gBlocks). Using Gibson assembly, DNA fragments were each cloned into the pT4 SB plasmid (gift from Zsuzsanna Iszvak), which in addition carried a GFP expression cassette to allow for enrichment of successful integration. HEK293T cells were electroporated with 4 μg pT4 transposon plasmid together with 1 μg of the SB100X transposase RNA using the 4D-Nucleofector (Lonza). After culturing the cells for 3 days, they were single-cell sorted for low GFP expression and colonies of single clones were expanded and sequenced for the correct integration of targets. To ensure low and comparable copy numbers of integrated DNA fragments, the four cell lines were transfected with identical amounts of DNA and transposase and sorted on the same day using an identical fluorescence-activated cell sorting (FACS) gating strategy (Figure S2A), enriching for low GFP expression levels.

Editing of patient-derived hURECs

After written informed consent (local institutional review board ethics vote no. EA4/066/21), patients donated fresh urine samples for hUREC cultivation. The isolation and characterization of hUREC used in this study have been described previously.[40,41] Cells were maintained in Advanced DMEM/F12 (Thermo Fisher Scientific) supplemented with 10% (v/v) fetal bovine serum (Sigma, catalog no. F7524), 1% P/S, 1 nM triiodothyronine, 10 ng/mL epidermal growth factor, 180 μM adenine, 25 ng/mL hydrocortisone, 1x GlutaMAX, and 10 mM HEPES, at 37°C in a humidified atmosphere

containing 5% CO_2. For genome editing, 2×10^5 hURECs per condition were electroporated with 2 μg total RNA (sgRNA:BE ratio of 1:3) using the 4D-Nucleofector system (Lonza). Cells were cultured for 2 days post-electroporation, after which they were harvested, and gDNA was extracted as previously described. To detect the *PKD1* c.9340C<T mutation and to exclude amplification of pseudogenes, long-range PCR spanning exons 26 to 34 using Q5 polymerase and specific primers (Table S1) was performed. Editing efficiency was assessed by Sanger sequencing of the PCR amplicons.

AAV production

Cbh_v5_AAV-ABE_N and Cbh_v5_AAV-ABE_C were a gift from David Liu (Addgene plasmids nos. 137177 and 137178) with the C-terminal part carrying sgRNA_45 (Table S2).[24] The duplexed oligos of the corresponding spacer sequence were annealed and ligated into the BsmBI-digested C-terminal plasmid. The AAV vectors used were constructed and packaged by the Charité Viral Core Facility or VectorBuilder. Cleanup was done using an iodixanol gradient centrifugation or cesium chloride; pAdDeltaF6 and pAAV2/8 were used as helper and REP/CAP plasmids.

Murine experiments

Mice initially generated on a mixed C57BL/6J × 129 background were backcrossed to C57BL/6J for at least eight generations ($N \geq 8$) to reduce residual genetic variability. Mice of both sexes were used. The mouse lines used in this study were previously described and include *Pkd1*$^{R2216W/fl}$ and *UBC-Cre-ERT2* and Ai14 reporter mice (Jax no. 007914).[23,42] The *Pkd1*$^{R2216W/fl}$;*UBC-Cre-ERT2* mice display Cre expression in the liver bile ducts and kidney proximal tubules upon tamoxifen induction. For monitoring of Cre delivery, Ai14 mice were injected with AAV8-Cre (2×10^{11} VG/mouse, single dose) at 5 weeks of age, and tdTomato expression was analyzed in kidney and liver 2 weeks later by flow cytometry and immunofluorescence. Deletion of the *Pkd1*-floxed allele was induced with tamoxifen between P28 and P42 followed by dual AAV8 split-ABE i.p. injection (one-time administration) at P49 with a total concentration of 8×10^{11} VG/mouse. The phenotype was analyzed 12 weeks post-treatment. We examined liver-to-body weight ratio and liver cystic index. Animal numbers for each study were determined by power calculations before initiation of the study. All animals used in this study were in accordance with scientific, human, and ethical principles and in compliance with animal welfare regulations approved by the Yale Institutional Animal Care and Use Committee or the Landesamt für Gesundheit und Soziales, Berlin.

Protein preparation and immunoblot analysis

Cultured cells were extracted and homogenized in an ice-cold homogenization buffer (250 mM sucrose and 10 mM triethanolamine, pH 8.45 containing protease inhibitors). The homogenates were then sonicated 5 times for 1 s each, followed by centrifugation at 1,000 × g for 10 min. Supernatant was analyzed as total lysate. Immunoblotting was performed using rabbit anti-HSP90 (Santa Cruz Biotechnology, catalog no. sc-7947, 1:5,000), mouse anti-PC1, 7E12 (Invitrogen, catalog no. mA5–15253, 1:500), rabbit anti-XBP1s (Abcam, catalog no. ab220783, 1:2,000), and mouse anti-β-actin (Sigma, catalog no. A2228, 1:5,000). PC1 and HSP90 were analyzed from the same samples resolved on a 4% gel. After membrane transfer, the blot was cut to allow different incubation conditions. sXBP1 and β-actin were resolved on a 10% gel and probed sequentially, using the same lysates as those used for PC1 and HSP90. Secondary antibodies included anti-mouse/rabbit horseradish peroxidase conjugates (1:2,000; Jackson ImmunoResearch Laboratories) and were incubated with the membrane for 1 h at room temperature. Thermo Scientific Pierce ECL Plus Western Blotting Substrate (Thermo Scientific, catalog no. 11527271) or SuperSignal West Femto Chemiluminescent Substrate (Thermo Fisher Scientific, catalog no. 34094) was used for chemiluminescence detection. The volume of individual immunoblot bands, in pixels, was determined by optical densitometry using ImageJ software (NIH).

Deglycosylation assay

For deglycosylation, 30 μg protein lysate was denatured in 10× Glycoprotein Denaturing Buffer (New England Biolabs, catalog no. B1704SVIAL, lot no. 0161707) at 60°C for 10 min and then cooled on ice. Samples were incubated with either Endo H (New England Biolabs, catalog no. P0702L, lot no. 1020316) in 10× GlycoBuffer 3 (catalog no. B1720SVIAL, lot no. 10048943) or PNGase F (New England Biolabs, catalog no. P0704S, lot no. 10226044) in 10× GlycoBuffer 2 (New England Biolabs, catalog no. B3704SVIAL, lot no. 0031609) supplemented with NP-40 (New England Biolabs, catalog no. B2704SVIAL, lot no. 0141609), at 37°C for 1 h. Before SDS-PAGE, samples were mixed with Bolt LDS Sample Buffer (4×) (Thermo Fisher, catalog no. B0007) and NuPAGE Sample Reducing Agent (10×) (Thermo Fisher, catalog no. NP0009) and then incubated at 60°C for 10 min and resolved on NuPAGE 3%–8% Tris-acetate gels (Thermo Fisher, catalog no. EA0378BOX).

Liver and kidney histological assessment

Mice were anesthetized by injecting ketamine/xylazine i.p. followed by cardiac perfusion with 1× PBS. The liver and kidney were then extracted, one part of which was snap frozen, and the remaining liver and kidney were fixed in 10% formalin for histological sectioning (5 μm) at the Research Histology Lab, Department of Comparative Medicine, Yale University. Hematoxylin and eosin sections thus obtained were imaged and scanned (4×) to measure the cystic index using a Nikon Eclipse TE2000-U microscope by CystAnalyser and MetaMorph software (Universal Imaging).[43,44]

Statistical analysis

Comparisons of three or more groups were performed using one-way ANOVA followed by Tukey's multiple group comparison post-hoc test. Comparison of two groups was performed using the two-tailed t test. A $p < 0.05$ was considered the threshold for statistical significance. Data are presented as the mean ± SD.

DATA AND CODE AVAILABILITY

The authors confirm that the data supporting the findings of this study are available within the article and its supplemental information or available from the corresponding authors upon reasonable request.

ACKNOWLEDGMENTS

M.M.K. was supported by the Emmy Noether Programme (grant no. KA5060/1-1) of the German Research Foundation (DFG) and is a participant in the BIH Charité Clinician Scientist Program funded by the Charité – Universitätsmedizin Berlin and the Berlin Institute of Health at Charité (BIH). A.I. was supported by the Add-on Fellowship for Interdisciplinary Life Science from the Joachim Herz Foundation. J.H. and R.S. receive funding from the German Research Foundation (DFG; project nos. HA 6908/4-1, HA 6908/7-1, HA 6908/8-1, HA 6908/12-1, and 539950728). J.H. and K.-U.E. are members of the European Reference Network for Rare Kidney Diseases (ERKNet). V.K. is a participant in the BIH Charité Clinician Scientist Program funded by the Charité – Universitätsmedizin Berlin and the Berlin Institute of Health at Charité (BIH) and supported by the Else Kröner-Fresenius-Stiftung (Else-Kröner Memorial Grant). R.B., F.H., and S.F. were supported by a DOD Investigator-Initiated Research Award to S.F. (GR114977). We thank the Viral Core Facility of the Charité – Universitätsmedizin Berlin for producing AAVs. We thank Lonnette Diggs in the George M. O'Brien Kidney Center at Yale (P30 DK079310) for blood urea nitrogen and creatinine measurements. We thank Claudia Diezemann for excellent technical assistance. The figures were created with BioRender.com.

AUTHOR CONTRIBUTIONS

A.I., R.B., D.E.Y., S.W., C.C., D.P., M.P., R.S., Q.M., I.V., and F.H. performed the experiments and analyzed the data. A.I., V.K., M.K., and M.M.K. wrote the original draft. S.K. performed the bioinformatic analysis. K.-U.E. advised on disease pathology. J.H. contributed patient information and provided expertise in human genetics. S.F., M.K., and M.M.K. acquired the funding, supervised the study, and analyzed the data. M.K., S.F., and M.M.K. conceptualized the study. All authors reviewed and edited the manuscript.

DECLARATION OF INTERESTS

A patent application related to this work has been filed.

DECLARATION OF GENERATIVE AI AND AI-ASSISTED TECHNOLOGIES IN THE WRITING PROCESS

During the preparation of this work the authors used AI-assisted tools (OpenAI GPT-5 mini) to improve readability and language. After using this tool, the authors reviewed and edited the content as needed and take full responsibility for the content of the publication.

SUPPLEMENTAL INFORMATION

Supplemental information can be found online at https://doi.org/10.1016/j.ymthe.2025.08.026.

REFERENCES

1. Bergmann, C., Guay-Woodford, L.M., Harris, P.C., Horie, S., Peters, D.J.M., and Torres, V.E. (2018). Polycystic kidney disease. Nat. Rev. Dis. Primers *4*, 50. https://doi.org/10.1038/s41572-018-0047-y.

2. Cornec-Le Gall, E., Alam, A., and Perrone, R.D. (2019). Autosomal dominant polycystic kidney disease. Lancet *393*, 919–935. https://doi.org/10.1016/S0140-6736(18)32782-X.

3. Torres, V.E., Ahn, C., Barten, T.R.M., Brosnahan, G., Cadnapaphornchai, M.A., Chapman, A.B., Cornec-Le Gall, E., Drenth, J.P.H., Gansevoort, R.T., Harris, P.C., et al. (2025). KDIGO 2025 clinical practice guideline for the evaluation, management, and treatment of autosomal dominant polycystic kidney disease (ADPKD): executive summary. Kidney Int. *107*, 234–254. https://doi.org/10.1016/j.kint.2024.07.010.

4. Sierks, D., Schönauer, R., Friedrich, A., Hantmann, E., de Fallois, J., Linder, N., Fischer, J., Herber, A., Bergmann, C., Berg, T., and Halbritter, J. (2022). Modelling polycystic liver disease progression using age-adjusted liver volumes and targeted mutational analysis. JHEP Rep. *4*, 100579. https://doi.org/10.1016/j.jhepr.2022.100579.

5. Harris, P.C., and Rossetti, S. (2010). Molecular diagnostics for autosomal dominant polycystic kidney disease. Nat. Rev. Nephrol. *6*, 197–206. https://doi.org/10.1038/nrneph.2010.18.

6. Trujillano, D., Bullich, G., Ossowski, S., Ballarín, J., Torra, R., Estivill, X., and Ars, E. (2014). Diagnosis of autosomal dominant polycystic kidney disease using efficient PKD1 and PKD2 targeted next-generation sequencing. Mol. Genet. Genomic Med. *2*, 412–421. https://doi.org/10.1002/mgg3.82.

7. Hopp, K., Ward, C.J., Hommerding, C.J., Nasr, S.H., Tuan, H.-F., Gainullin, V.G., Rossetti, S., Torres, V.E., and Harris, P.C. (2012). Functional polycystin-1 dosage governs autosomal dominant polycystic kidney disease severity. J. Clin. Invest. *122*, 4257–4273. https://doi.org/10.1172/JCI64313.

8. Lantinga-van Leeuwen, I.S., Dauwerse, J.G., Baelde, H.J., Leonhard, W.N., van de Wal, A., Ward, C.J., Verbeek, S., Deruiter, M.C., Breuning, M.H., de Heer, E., and Peters, D.J.M. (2004). Lowering of Pkd1 expression is sufficient to cause polycystic kidney disease. Hum. Mol. Genet. *13*, 3069–3077. https://doi.org/10.1093/hmg/ddh336.

9. Qian, F., Watnick, T.J., Onuchic, L.F., and Germino, G.G. (1996). The molecular basis of focal cyst formation in human autosomal dominant polycystic kidney disease type I. Cell *87*, 979–987. https://doi.org/10.1016/s0092-8674(00)81793-6.

10. Dong, K., Zhang, C., Tian, X., Coman, D., Hyder, F., Ma, M., and Somlo, S. (2021). Renal plasticity revealed through reversal of polycystic kidney disease in mice. Nat. Genet. *53*, 1649–1663. https://doi.org/10.1038/s41588-021-00946-4.

11. Lakhia, R., Ramalingam, H., Chang, C.-M., Cobo-Stark, P., Biggers, L., Flaten, A., Alvarez, J., Valencia, T., Wallace, D.P., Lee, E.C., and Patel, V. (2022). PKD1 and PKD2 mRNA cis-inhibition drives polycystic kidney disease progression. Nat. Commun. *13*, 4765. https://doi.org/10.1038/s41467-022-32543-2.

12. Kurbegovic, A., Pacis, R.C., and Trudel, M. (2023). Modeling Pkd1 gene-targeted strategies for correction of polycystic kidney disease. Mol. Ther. Methods Clin. Dev. *29*, 366–380. https://doi.org/10.1016/j.omtm.2023.03.016.

13. Komor, A.C., Kim, Y.B., Packer, M.S., Zuris, J.A., and Liu, D.R. (2016). Programmable editing of a target base in genomic DNA without double-stranded DNA cleavage. Nature *533*, 420–424. https://doi.org/10.1038/nature17946.

14. Gaudelli, N.M., Komor, A.C., Rees, H.A., Packer, M.S., Badran, A.H., Bryson, D.I., and Liu, D.R. (2017). Programmable base editing of A·T to G·C in genomic DNA without DNA cleavage. Nature *551*, 464–471. https://doi.org/10.1038/nature24644.

15. Tong, H., Wang, X., Liu, Y., Liu, N., Li, Y., Luo, J., Ma, Q., Wu, D., Li, J., Xu, C., and Yang, H. (2023). Programmable A-to-Y base editing by fusing an adenine base editor with an N-methylpurine DNA glycosylase. Nat. Biotechnol. *41*, 1080–1084. https://doi.org/10.1038/s41587-022-01595-6.

16. Kurt, I.C., Zhou, R., Iyer, S., Garcia, S.P., Miller, B.R., Langner, L.M., Grünewald, J., and Joung, J.K. (2021). CRISPR C-to-G base editors for inducing targeted DNA transversions in human cells. Nat. Biotechnol. *39*, 41–46. https://doi.org/10.1038/s41587-020-0609-x.

17. Koblan, L.W., Erdos, M.R., Wilson, C., Cabral, W.A., Levy, J.M., Xiong, Z.-M., Tavarez, U.L., Davison, L.M., Gete, Y.G., Mao, X., et al. (2021). In vivo base editing rescues Hutchinson-Gilford progeria syndrome in mice. Nature *589*, 608–614. https://doi.org/10.1038/s41586-020-03086-7.

18. Villiger, L., Grisch-Chan, H.M., Lindsay, H., Ringnalda, F., Pogliano, C.B., Allegri, G., Fingerhut, R., Häberle, J., Matos, J., Robinson, M.D., et al. (2018). Treatment of a metabolic liver disease by in vivo genome base editing in adult mice. Nat. Med. *24*, 1519–1525. https://doi.org/10.1038/s41591-018-0209-1.

19. Rees, H.A., and Liu, D.R. (2018). Base editing: precision chemistry on the genome and transcriptome of living cells. Nat. Rev. Genet. *19*, 770–788. https://doi.org/10.1038/s41576-018-0059-1.

20. Gaudelli, N.M., Lam, D.K., Rees, H.A., Solá-Esteves, N.M., Barrera, L.A., Born, D.A., Edwards, A., Gehrke, J.M., Lee, S.J., Liquori, A.J., et al. (2020). Directed evolution of adenine base editors with increased activity and therapeutic application. Nat. Biotechnol. *38*, 892–900. https://doi.org/10.1038/s41587-020-0491-6.

21. Vujic, M., Heyer, C.M., Ars, E., Hopp, K., Markoff, A., Orndal, C., Rudenhed, B., Nasr, S.H., Torres, V.E., Torra, R., et al. (2010). Incompletely penetrant PKD1 alleles mimic the renal manifestations of ARPKD. J. Am. Soc. Nephrol. *21*, 1097–1102. https://doi.org/10.1681/ASN.2009101070.

22. Fedeles, S.V., So, J.-S., Shrikhande, A., Lee, S.H., Gallagher, A.-R., Barkauskas, C.E., Somlo, S., and Lee, A.-H. (2015). Sec63 and Xbp1 regulate IRE1α activity and polycystic disease severity. J. Clin. Invest. *125*, 1955–1967. https://doi.org/10.1172/JCI78863.

23. Krappitz, M., Bhardwaj, R., Dong, K., Staudner, T., Yilmaz, D.E., Pioppini, C., Westergerling, P., Ruemmele, D., Hollmann, T., Nguyen, T.A., et al. (2023). XBP1

Activation Reduces Severity of Polycystic Kidney Disease due to a Nontruncating Polycystin-1 Mutation in Mice. J. Am. Soc. Nephrol. *34*, 110–121. https://doi.org/10.1681/ASN.2021091180.

24. Levy, J.M., Yeh, W.H., Pendse, N., Davis, J.R., Hennessey, E., Butcher, R., Koblan, L.W., Comander, J., Liu, Q., and Liu, D.R. (2020). Cytosine and adenine base editing of the brain, liver, retina, heart and skeletal muscle of mice via adeno-associated viruses. Nat. Biomed. Eng. *4*, 97–110. https://doi.org/10.1038/s41551-019-0501-5.

25. Bae, S., Park, J., and Kim, J.-S. (2014). Cas-OFFinder: a fast and versatile algorithm that searches for potential off-target sites of Cas9 RNA-guided endonucleases. Bioinformatics *30*, 1473–1475. https://doi.org/10.1093/bioinformatics/btu048.

26. Furusho, T., Das, R., Hakui, H., Sairavi, A., Adachi, K., Galbraith-Liss, M.S., Rajagopal, P., Horikawa, M., Luo, S., Li, L., et al. (2024). Enhancing gene transfer to renal tubules and podocytes by context-dependent selection of AAV capsids. Nat. Commun. *15*, 10728. https://doi.org/10.1038/s41467-024-54475-9.

27. Rosales, A., Blondel, L.O., Hull, J., Gao, Q., Aykun, N., Peek, J.L., Vargas, A., Fergione, S., Song, M., Wilson, M.H., et al. (2025). Evolving adeno-associated viruses for gene transfer to the kidney via cross-species cycling of capsid libraries. Nat. Biomed. Eng. *9*, 1086–1100. https://doi.org/10.1038/s41551-024-01341-0.

28. Reichart, D., Newby, G.A., Wakimoto, H., Lun, M., Gorham, J.M., Curran, J.J., Raguram, A., DeLaughter, D.M., Conner, D.A., Marsiglia, J.D.C., et al. (2023). Efficient in vivo genome editing prevents hypertrophic cardiomyopathy in mice. Nat. Med. *29*, 412–421. https://doi.org/10.1038/s41591-022-02190-7.

29. Rothgangl, T., Dennis, M.K., Lin, P.J.C., Oka, R., Witzigmann, D., Villiger, L., Qi, W., Hruzova, M., Kissling, L., Lenggenhager, D., et al. (2021). In vivo adenine base editing of PCSK9 in macaques reduces LDL cholesterol levels. Nat. Biotechnol. *39*, 949–957. https://doi.org/10.1038/s41587-021-00933-4.

30. Brooks, D.L., Whittaker, M.N., Said, H., Dwivedi, G., Qu, P., Musunuru, K., Ahrens-Nicklas, R.C., Alameh, M.-G., and Wang, X. (2024). A base editing strategy using mRNA-LNPs for in vivo correction of the most frequent phenylketonuria variant. HGG Adv. *5*, 100253. https://doi.org/10.1016/j.xhgg.2023.100253.

31. Koblan, L.W., Doman, J.L., Wilson, C., Levy, J.M., Tay, T., Newby, G.A., Maianti, J.P., Raguram, A., and Liu, D.R. (2018). Improving cytidine and adenine base editors by expression optimization and ancestral reconstruction. Nat. Biotechnol. *36*, 843–846. https://doi.org/10.1038/nbt.4172.

32. Anzalone, A.V., Randolph, P.B., Davis, J.R., Sousa, A.A., Koblan, L.W., Levy, J.M., Chen, P.J., Wilson, C., Newby, G.A., Raguram, A., and Liu, D.R. (2019). Search-and-replace genome editing without double-strand breaks or donor DNA. Nature *576*, 149–157. https://doi.org/10.1038/s41586-019-1711-4.

33. Alves, C.R.R., Ha, L.L., Yaworski, R., Sutton, E.R., Lazzarotto, C.R., Christie, K.A., Reilly, A., Beauvais, A., Doll, R.M., de la Cruz, D., et al. (2024). Optimization of base editors for the functional correction of SMN2 as a treatment for spinal muscular atrophy. Nat. Biomed. Eng. *8*, 118–131. https://doi.org/10.1038/s41551-023-01132-z.

34. Richter, M.F., Zhao, K.T., Eton, E., Lapinaite, A., Newby, G.A., Thuronyi, B.W., Wilson, C., Koblan, L.W., Zeng, J., Bauer, D.E., et al. (2020). Phage-assisted evolution of an adenine base editor with improved Cas domain compatibility and activity. Nat. Biotechnol. *38*, 883–891. https://doi.org/10.1038/s41587-020-0453-z.

35. Walton, R.T., Christie, K.A., Whittaker, M.N., and Kleinstiver, B.P. (2020). Unconstrained genome targeting with near-PAMless engineered CRISPR-Cas9 variants. Science *368*, 290–296. https://doi.org/10.1126/science.aba8853.

36. Baert, L. (1978). Hereditary polycystic kidney disease (adult form): a microdissection study of two cases at an early stage of the disease. Kidney Int. *13*, 519–525. https://doi.org/10.1038/ki.1978.75.

37. Nishio, S., Hatano, M., Nagata, M., Horie, S., Koike, T., Tokuhisa, T., and Mochizuki, T. (2005). Pkd1 regulates immortalized proliferation of renal tubular epithelial cells through p53 induction and JNK activation. J. Clin. Invest. *115*, 910–918. https://doi.org/10.1172/JCI22850.

38. Clement, K., Rees, H., Canver, M.C., Gehrke, J.M., Farouni, R., Hsu, J.Y., Cole, M.A., Liu, D.R., Joung, J.K., Bauer, D.E., and Pinello, L. (2019). CRISPResso2 provides accurate and rapid genome editing sequence analysis. Nat. Biotechnol. *37*, 224–226. https://doi.org/10.1038/s41587-019-0032-3.

39. Mátés, L., Chuah, M.K.L., Belay, E., Jerchow, B., Manoj, N., Acosta-Sanchez, A., Grzela, D.P., Schmitt, A., Becker, K., Matrai, J., et al. (2009). Molecular evolution of a novel hyperactive Sleeping Beauty transposase enables robust stable gene transfer in vertebrates. Nat. Genet. *41*, 753–761. https://doi.org/10.1038/ng.343.

40. Yealland, G., Jevtic, M., Eckardt, K.-U., and Schueler, M. (2023). Modeling ciliopathies in patient-derived primary cells. Methods Cell Biol. *176*, 139–158. https://doi.org/10.1016/bs.mcb.2023.02.016.

41. Zhou, T., Benda, C., Dunzinger, S., Huang, Y., Ho, J.C., Yang, J., Wang, Y., Zhang, Y., Zhuang, Q., Li, Y., et al. (2012). Generation of human induced pluripotent stem cells from urine samples. Nat. Protoc. *7*, 2080–2089. https://doi.org/10.1038/nprot.2012.115.

42. Ma, M., Tian, X., Igarashi, P., Pazour, G.J., and Somlo, S. (2013). Loss of cilia suppresses cyst growth in genetic models of autosomal dominant polycystic kidney disease. Nat. Genet. *45*, 1004–1012. https://doi.org/10.1038/ng.2715.

43. Shibazaki, S., Yu, Z., Nishio, S., Tian, X., Thomson, R.B., Mitobe, M., Louvi, A., Velazquez, H., Ishibe, S., Cantley, L.G., et al. (2008). Cyst formation and activation of the extracellular regulated kinase pathway after kidney specific inactivation of Pkd1. Hum. Mol. Genet. *17*, 1505–1516. https://doi.org/10.1093/hmg/ddn039.

44. Cordido, A., Cernadas, E., Fernández-Delgado, M., and García-González, M.A. (2020). CystAnalyser: A new software tool for the automatic detection and quantification of cysts in Polycystic Kidney and Liver Disease, and other cystic disorders. PLoS Comput. Biol. *16*, e1008337.

YMTHE, Volume *33*

Supplemental Information

In vivo base editing reduces liver cysts in autosomal dominant polycystic kidney disease

Antonia Ibel, Rishi Bhardwaj, Duygu Elif Yilmaz, Shuhan Kong, Sarah Wendlinger, Carlos Cordero, Dimitra Papaioannou, Maria Papazian, Ria Schönauer, Qiyao Meng, Kai-Uwe Eckardt, Fatima Hassan, Isabel Volpe, Verena Klämbt, Jan Halbritter, Sorin Fedeles, Matteus Krappitz, and Michael M. Kaminski

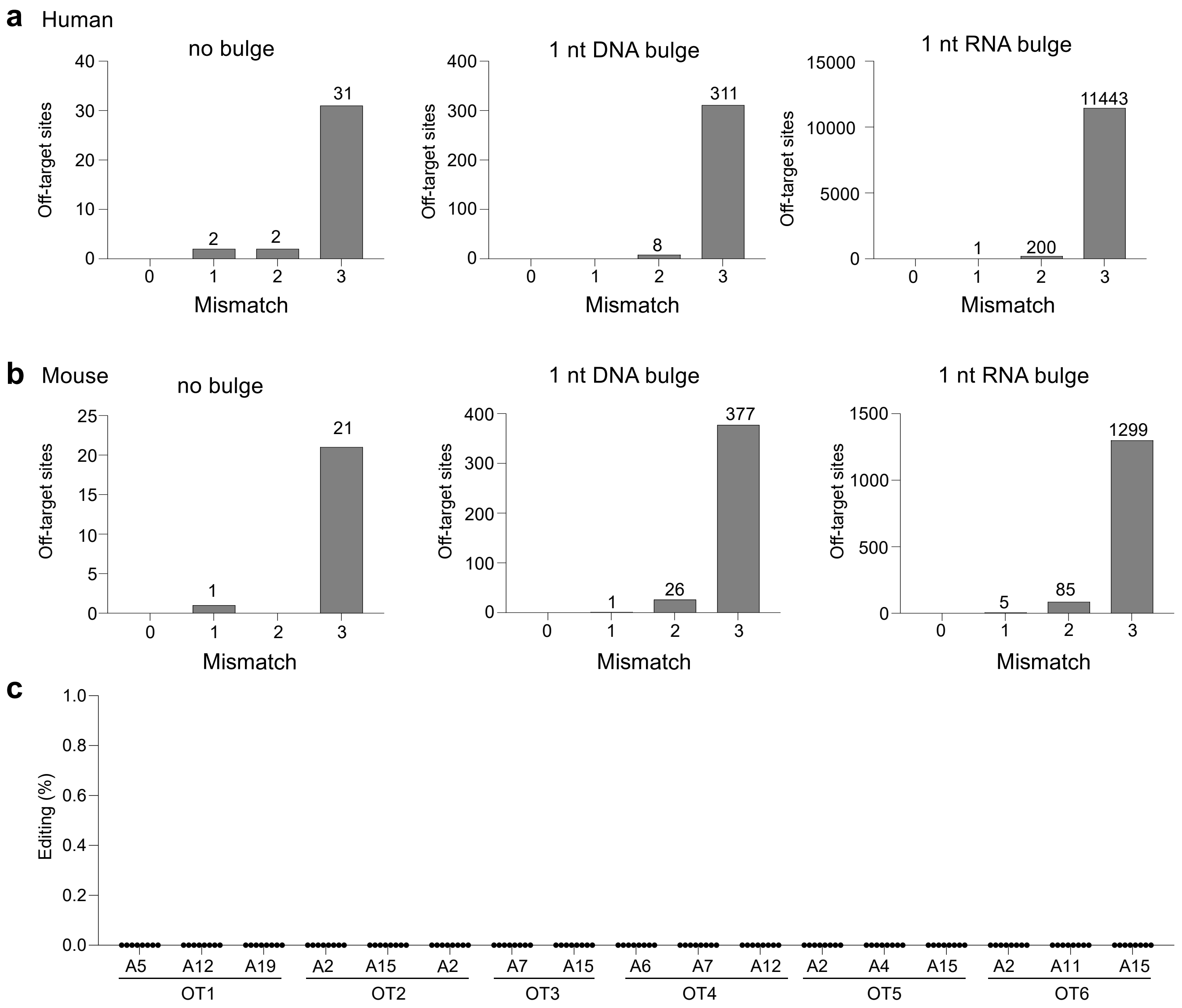

Figure S1: Off-target analysis for RW correcting human and mouse sgRNAs. a, Number of CasOFFinder annotated putative off-target sites in the human genome for sgRNA_20 (for SpCas9 and a NGG PAM). Predicted off-target sites for one and two mismatch sites (without bulges) were identified within *PKD1* pseudogenes**. b,** Number of CasOFFinder annotated putative off-target sites in the mouse genome for sgRNA_45 (for SpCas9 and a NGG PAM). The one site predicted for one mismatch (no bulge) marks the target site. **c,** *In vivo* editing efficiency (y-axis) for the top 6 predicted mouse off-target sites (OT) as determined by targeted amplicon sequencing of gDNA isolated from liver tissue at all possible adenines (A).

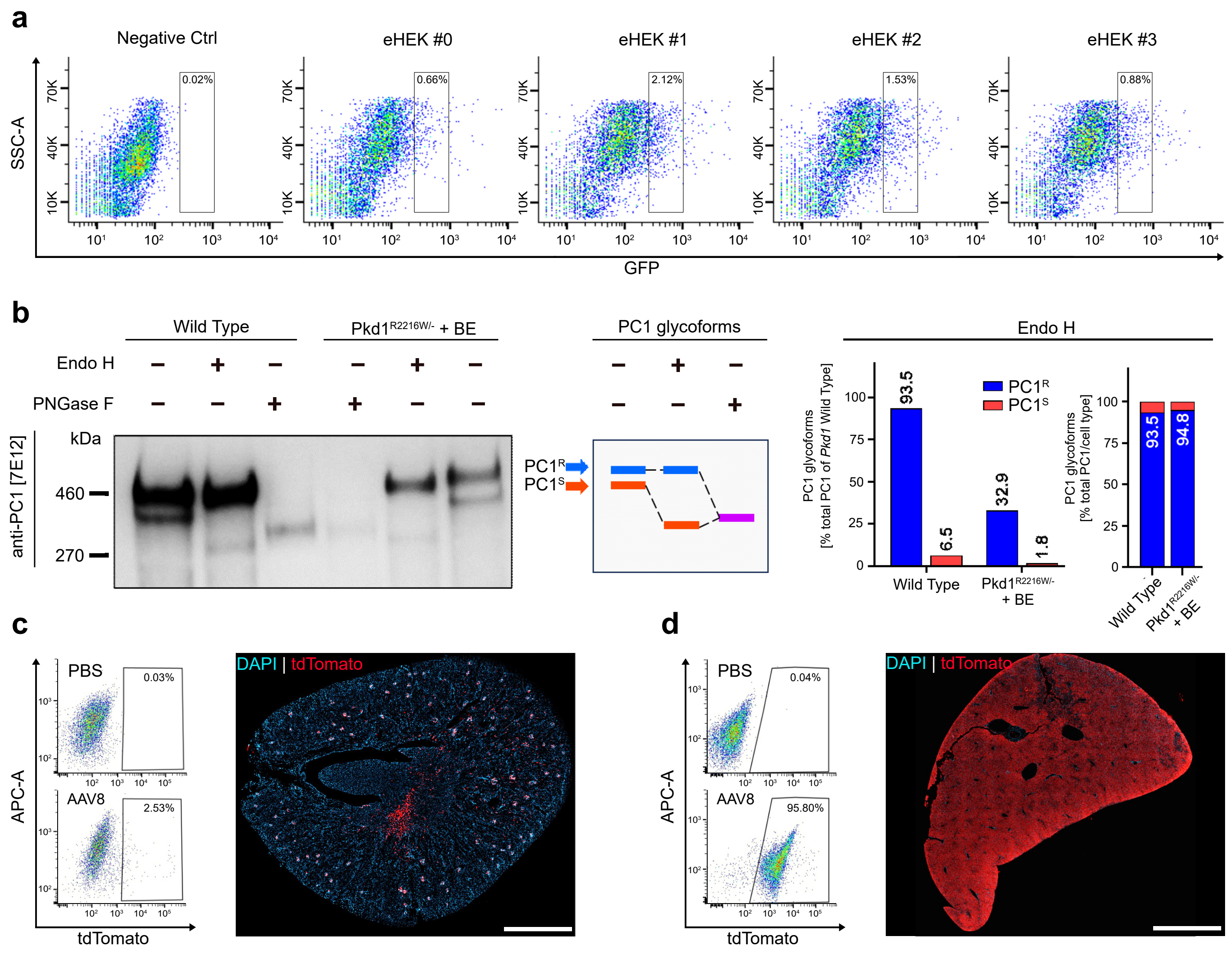

Figure S2: Quality control for engineered HEK293T cell lines and RTEC editing and assessment of *in vivo* AAV8 transduction efficiency in the kidney and liver. a, FACS plots illustrating the gating strategy used for sorting the four engineered HEK293T cell lines (eHEK #0-#3). Transfection and sorting were performed on the same day, applying identical gates to select for cells with medium-to-low expression levels to ensure consistent and low copy number integration across all four lines. **b,** Deglycosylation analysis of PC1 in base edited and control RTEC cells reveals comparable amounts of mature Endo H-resistant (PC1^R; blue) and immature Endo H-sensitive (PC1^S, red) glycoforms. PNGase F served as a positive control for complete deglycosylation (violet). **c,** Systemic AAV8-Cre delivery (1x10^{13}VG/kg) in Ai14 reporter mice show limited transduction in the kidney. Left panel: flow cytometry analysis of tdTomato expresion in dissociated kidney cells. Top left plot shows kidney cells from a PBS-injected control mouse, while the bottom left plot shows kidney cells from an AAV8-Cre–injected mouse. Right panel: representative immunofluorescence image of a coronal kidney section from an AAV8-Cre–injected mouse. tdTomato expression is shown in red; nuclei are counterstained with DAPI (turquoise). Scale bar = 1000 µm. **d,** Systemic AAV8-Cre delivery (1x10^{13} VG/kg) in Ai14 reporter mice show successful transduction in the liver. Left panel: flow cytometry analysis of tdTomato expression in dissociated liver cells. Top left plot shows liver cells from a PBS-injected control mouse; the bottom left plot shows liver cells from an AAV8-Cre–injected mouse. Right panel: representative immunofluorescence image of a liver section from an AAV8-Cre–injected mouse. tdTomato expression is shown in red; nuclei are counterstained with DAPI (turquoise). Scale bar = 1000 µm.

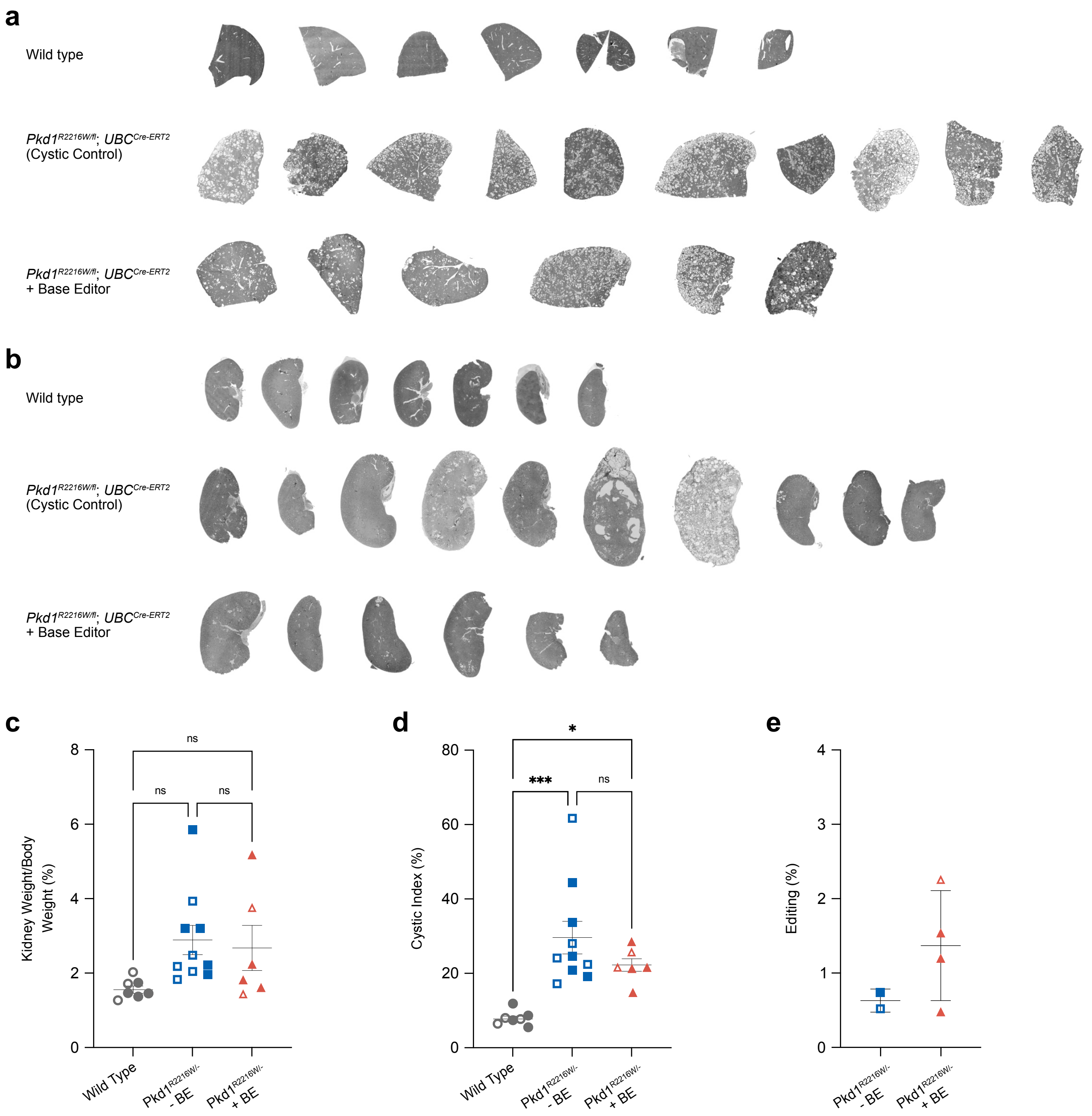

Figure S3: *In vivo* base editing shows no influence on kidney phenotype in the RW knock-in mouse model. a, Microscopic overview images of liver tissue from the indicated conditions from all animals. **b,** Microscopic overview images of kidney tissue from the indicated conditions from all animals. **c, d,** Quantification of kidney-to-body weight ratios (**c**) and kidney cystic indices (**d**) between wild-type mice (n=7), untreated RW knock-in mice (n=10) and base editor (BE) treated RW knock-in mice (n=6). **e,** Quantification of editing efficiency in gDNA isolated from the kidney of the untreated RW knock-in mice (n=2) and base editor (BE) treated RW knock-in mice (n=4). **c, d, e,** Data points represent independent biological replicates with means ± SD, $***p < 0.001$, $*p < 0.05$, ns: not significant. Filled data points indicate male mice, hollow once female mice.

Table S1: Primer sequences for targeted amplicon sequencing (TAS) and long-range PCR (LR-PCR). For TAS, PCR-1 was used for target locus–specific amplification and PCR-2 for addition of barcodes and Illumina adapter sequences. All sequences are shown in the 5′→3′ orientation.

Table S1

Primer Name	Primer Sequence (5'-3')	Used for
16SrRNA_63f fwd/fwd_TAS	TCGTCGGCAGCGTCAGATGTGTATAAGAGACAGCAGGCCTAACACATGCAAGTC	PCR-1
16SrRNA_1387f rev/rev_TAS	GTCTCGTGGGCTCGGAGATGTGTATAAGAGACAGGCCTTGTACACTCCGCCC	PCR-1
16SrRNA_1387r rev/fwd_TAS	TCGTCGGCAGCGTCAGATGTGTATAAGAGACAGGGGCGGAGTGTACAAGGC	PCR-1
AmpR rev_TAS	GTCTCGTGGGCTCGGAGATGTGTATAAGAGACAGGCTATGTGGCGCGGTATTAT	PCR-1
AmpR fwd_TAS	TCGTCGGCAGCGTCAGATGTGTATAAGAGACAGATAATACCGCGCCACATAGC	PCR-1
LucNrev rev_TAS	GTCTCGTGGGCTCGGAGATGTGTATAAGAGACAGGGAGAGCAACTGCATAAGG	PCR-1
LucNrev fwd_TAS	TCGTCGGCAGCGTCAGATGTGTATAAGAGACAGCCTTATGCAGTTGCTCTCC	PCR-1
Partial-P5 rev_TAS	GTCTCGTGGGCTCGGAGATGTGTATAAGAGACAGTCTCGGTGGTCGCCGTATCATT	PCR-1
Partial-P5 fwd_TAS	TCGTCGGCAGCGTCAGATGTGTATAAGAGACAGAATGATACGGCGACCACCGAGA	PCR-1
pUC19-fwd/rev_TAS	GTCTCGTGGGCTCGGAGATGTGTATAAGAGACAGCTTAGACGTCAGGTGGCACTT	PCR-1
pUC19-fwd/fwd_TAS	TCGTCGGCAGCGTCAGATGTGTATAAGAGACAGAAGTGCCACCTGACGTCTAAG	PCR-1
TSO enrichment rev_TAS	GTCTCGTGGGCTCGGAGATGTGTATAAGAGACAGAAGCAGTGGTATCAACGCAGAGT	PCR-1
TSO enrichment fwd_TAS	TCGTCGGCAGCGTCAGATGTGTATAAGAGACAGACTCTGCGTTGATACCACTGCTT	PCR-1
CMV Forward rev_TAS	GTCTCGTGGGCTCGGAGATGTGTATAAGAGACAGCACGCCTACCGCCCATTTGCG	PCR-1
CMV Forward fwd_TAS	TCGTCGGCAGCGTCAGATGTGTATAAGAGACAGCGCAAATGGGCGGTAGGCGTG	PCR-1
VR Primer rev_TAS	GTCTCGTGGGCTCGGAGATGTGTATAAGAGACAGGCTCACTCAAAGGCGGTAAT	PCR-1
VR Primer fwd_TAS	TCGTCGGCAGCGTCAGATGTGTATAAGAGACAGATTACCGCCTTTGAGTGAGC	PCR-1
oAI411_LucNrev_fwd	TCGTCGGCAGCGTCAGATGTGTATAAGAGACAGTATGCAGTTGCTCTCCGCACT	PCR-1
oAI411_AmpR_rev	GTCTCGTGGGCTCGGAGATGTGTATAAGAGACAGTATGTGGCGCGGTATTATCGTG	PCR-1
oAI411_AmpR_fwd	TCGTCGGCAGCGTCAGATGTGTATAAGAGACAGCACGATAATACCGCGCCACATA	PCR-1
oAI411_16S rRNA Primer 1387r_rev	GTCTCGTGGGCTCGGAGATGTGTATAAGAGACAGTTGTACACTCCGCCCACCGT	PCR-1
oAI411_16S rRNA Primer 1387r_fwd	TCGTCGGCAGCGTCAGATGTGTATAAGAGACAGACGGTGGGCGGAGTGTACAA	PCR-1
oAI411_16S rRNA Primer 63f_rev	GTCTCGTGGGCTCGGAGATGTGTATAAGAGACAGGCATGTGTTAGGCCTGTCCTG	PCR-1
oAI412_Tso_Enrich_fwd	TCGTCGGCAGCGTCAGATGTGTATAAGAGACAGGTTGATACCACTGCTTCACG	PCR-1
oAI412_pUC19_rev	GTCTCGTGGGCTCGGAGATGTGTATAAGAGACAGTTAGACGTCAGGTGGCACTTCT	PCR-1
oAI412_LucNrev_fwd	TCGTCGGCAGCGTCAGATGTGTATAAGAGACAGCCTGGCTGCCTTATGCAGTT	PCR-1
oAI412_AmpR_rev	GTCTCGTGGGCTCGGAGATGTGTATAAGAGACAGTCAGCCGTGAGCTATGTGG	PCR-1
oAI412_AmpR_fwd	TCGTCGGCAGCGTCAGATGTGTATAAGAGACAGCCACATAGCTCACGGCTGA	PCR-1
oAI412_16S rRNA Primer 1387r_rev	GTCTCGTGGGCTCGGAGATGTGTATAAGAGACAGGCTGTGCCTTGTACACTCCG	PCR-1
oAI412_16S rRNA Primer 1387r_fwd	TCGTCGGCAGCGTCAGATGTGTATAAGAGACAGCGGAGTGTACAAGGCACAGC	PCR-1
oAI412_16S rRNA Primer 63f_rev	GTCTCGTGGGCTCGGAGATGTGTATAAGAGACAGTGTGTTAGGCCTGACCCCAC	PCR-1
oAI412_16S rRNA Primer 63f_fwd	TCGTCGGCAGCGTCAGATGTGTATAAGAGACAGGTGGGGTCAGGCCTAACACA	PCR-1
oAI412_mCherry_rev	GTCTCGTGGGCTCGGAGATGTGTATAAGAGACAGTTCTGCATTACGGGGCGTCC	PCR-1
oAI413_LucNrev_fwd	TCGTCGGCAGCGTCAGATGTGTATAAGAGACAGCTTATGCAGTTGCTCTCCGGAT	PCR-1
oAI413_AmpR_rev	GTCTCGTGGGCTCGGAGATGTGTATAAGAGACAGAAGGCTTCACGCAGCTATGTG	PCR-1
oAI413_AmpR_fwd	TCGTCGGCAGCGTCAGATGTGTATAAGAGACAGCACATAGCTGCGTGAAGCCTT	PCR-1
oAI413_16S rRNA 1387_rev	GTCTCGTGGGCTCGGAGATGTGTATAAGAGACAGTAAGACGCCTTGTACACTCCG	PCR-1
oAI413_16S rRNA 1387_fwd	TCGTCGGCAGCGTCAGATGTGTATAAGAGACAGCGGAGTGTACAAGGCGTCTTA	PCR-1
oAI413_16S rRNA 63f_rev	GTCTCGTGGGCTCGGAGATGTGTATAAGAGACAGGGAAGACTTGCATGTGTTAGGC	PCR-1
oAI413_16S rRNA 63f_fwd	TCGTCGGCAGCGTCAGATGTGTATAAGAGACAGGCCTAACACATGCAAGTCTTCC	PCR-1
oAI413_mCherry_rev	GTCTCGTGGGCTCGGAGATGTGTATAAGAGACAGTTCTTCTGCATTACGGGGCAC	PCR-1
oAI414_LucNrev_fwd	TCGTCGGCAGCGTCAGATGTGTATAAGAGACAGACGGGCCTTATGCAGTTGCT	PCR-1
oAI414_AmpR_rev	GTCTCGTGGGCTCGGAGATGTGTATAAGAGACAGACAGCTATGTGGCGCGGTAT	PCR-1
oAI414_AmpR_fwd	TCGTCGGCAGCGTCAGATGTGTATAAGAGACAGATACCGCGCCACATAGCTGT	PCR-1
oAI414_16S rRNA 1387_rev	GTCTCGTGGGCTCGGAGATGTGTATAAGAGACAGTGAAGCCTTGTACACTCCGC	PCR-1
oAI414_16S rRNA 1387_fwd	TCGTCGGCAGCGTCAGATGTGTATAAGAGACAGGCGGAGTGTACAAGGCTTCA	PCR-1
oAI414_16S rRNA Primer 63f_rev	GTCTCGTGGGCTCGGAGATGTGTATAAGAGACAGCATGTGTTAGGCCTGACCGT	PCR-1
oAI414_16S rRNA Primer 63f_fwd	TCGTCGGCAGCGTCAGATGTGTATAAGAGACAGACGGTCAGGCCTAACACATG	PCR-1
oAI414_mCherry_rev	GTCTCGTGGGCTCGGAGATGTGTATAAGAGACAGTCTTCTGCATTACGGGGCTC	PCR-1
Pkd1_TAS R2216W Fwd	TCGTCGGCAGCGTCAGATGTGTATAAGAGACAGTGTAGGGAGCCTGAAGTGGA	PCR-1
Pkd1_TAS R2216W Rev	GTCTCGTGGGCTCGGAGATGTGTATAAGAGACAGCCTGCGTGTCTGACCATACC	PCR-1
N701	CAAGCAGAAGACGGCATACGAGATTCGCCTTAGTCTCGTGGGCTC	PCR-2
N702	CAAGCAGAAGACGGCATACGAGATCTAGTACGGTCTCGTGGGCTC	PCR-2
N703	CAAGCAGAAGACGGCATACGAGATTTCTGCCT GTCTCGTGGGCTC	PCR-2
N704	CAAGCAGAAGACGGCATACGAGATGCTCAGGAGTCTCGTGGGCTC	PCR-2
N705	CAAGCAGAAGACGGCATACGAGATAGGAGTCCGTCTCGTGGGCTC	PCR-2
N706	CAAGCAGAAGACGGCATACGAGATCATGCCTAGTCTCGTGGGCTC	PCR-2
N707	CAAGCAGAAGACGGCATACGAGATGTAGAGAGGTCTCGTGGGCTC	PCR-2
N710	CAAGCAGAAGACGGCATACGAGATCAGCCTCGGTCTCGTGGGCTC	PCR-2
N711	CAAGCAGAAGACGGCATACGAGATTGCCTCTTGTCTCGTGGGCTC	PCR-2
N712	CAAGCAGAAGACGGCATACGAGATTCCTCTACGTCTCGTGGGCTC	PCR-2
N714	CAAGCAGAAGACGGCATACGAGATTCATGAGCGTCTCGTGGGCTC	PCR-2
N715	CAAGCAGAAGACGGCATACGAGATCCTGAGATGTCTCGTGGGCTC	PCR-2
S502	AATGATACGGCGACCACCGAGATCTACACCTCTCTATTCGTCGGCAGCG	PCR-2
S503	AATGATACGGCGACCACCGAGATCTACACTATCCTCTTCGTCGGCAGCG	PCR-2
S505	AATGATACGGCGACCACCGAGATCTACACGTAAGGAGTCGTCGGCAGCG	PCR-2
S506	AATGATACGGCGACCACCGAGATCTACACACTGCATATCGTCGGCAGCG	PCR-2
S507	AATGATACGGCGACCACCGAGATCTACACAAGGAGTATCGTCGGCAGCG	PCR-2

Table S1 (continued)

Primer Name	Primer Sequence (5'-3')	Used for
S508	AATGATACGGCGACCACCGAGATCTACACCTAAGCCTTCGTCGGCAGCG	PCR-2
S510	AATGATACGGCGACCACCGAGATCTACACCGTCTAATTCGTCGGCAGCG	PCR-2
S511	AATGATACGGCGACCACCGAGATCTACACTCTCTCCGTCGTCGGCAGCG	PCR-2
N716	CAAGCAGAAGACGGCATACGAGATTAGCGAGTGTCTCGTGGGCTCGG	PCR-2
N718	CAAGCAGAAGACGGCATACGAGATGTAGCTCCGTCTCGTGGGCTCGG	PCR-2
N719	CAAGCAGAAGACGGCATACGAGATTACTACGCGTCTCGTGGGCTCGG	PCR-2
N720	CAAGCAGAAGACGGCATACGAGATAGGCTCCGGTCTCGTGGGCTCGG	PCR-2
N721	CAAGCAGAAGACGGCATACGAGATGCAGCGTAGTCTCGTGGGCTCGG	PCR-2
N722	CAAGCAGAAGACGGCATACGAGATCTGCGCATGTCTCGTGGGCTCGG	PCR-2
N723	CAAGCAGAAGACGGCATACGAGATGAGCGCTAGTCTCGTGGGCTCGG	PCR-2
N724	CAAGCAGAAGACGGCATACGAGATCGCTCAGTGTCTCGTGGGCTCGG	PCR-2
N726	CAAGCAGAAGACGGCATACGAGATGTCTTAGGGTCTCGTGGGCTCGG	PCR-2
N727	CAAGCAGAAGACGGCATACGAGATACTGATCGGTCTCGTGGGCTCGG	PCR-2
N728	CAAGCAGAAGACGGCATACGAGATTAGCTGCAGTCTCGTGGGCTCGG	PCR-2
N729	CAAGCAGAAGACGGCATACGAGATGACGTCGAGTCTCGTGGGCTCGG	PCR-2
S513	AATGATACGGCGACCACCGAGATCTACACTCGACTAGTCGTCGGCAGCGTC	PCR-2
S515	AATGATACGGCGACCACCGAGATCTACACTTCTAGCTTCGTCGGCAGCGTC	PCR-2
S516	AATGATACGGCGACCACCGAGATCTACACCCTAGAGTTCGTCGGCAGCGTC	PCR-2
S517	AATGATACGGCGACCACCGAGATCTACACGCGTAAGATCGTCGGCAGCGTC	PCR-2
S518	AATGATACGGCGACCACCGAGATCTACACCTATTAAGTCGTCGGCAGCGTC	PCR-2
S520	AATGATACGGCGACCACCGAGATCTACACAAGGCTATTCGTCGGCAGCGTC	PCR-2
S521	AATGATACGGCGACCACCGAGATCTACACGAGCCTTATCGTCGGCAGCGTC	PCR-2
S522	AATGATACGGCGACCACCGAGATCTACACTTATGCGATCGTCGGCAGCGTC	PCR-2
PKD1_c.9320_Exon25_fwd	TCGTGCCCCCAAGCCATGT	LR-PCR
PKD1_c.9320_exon34_rev	AGCCGGACACTCACAGGCT	LR-PCR

Table S2: Guide RNA (gRNA) sequences used for correction of the indicated variants via base editing. Sequences are shown in the 5′→3′ orientation, excluding the PAM sequence.

Table S2

**gRNA Name**	**Mutation**	**gRNA Sequence 5´-3´without PAM**	**Species**	**Gene**
gRNA_1	c.412C>T	CCCATCACGGCAGCCACGCC	human	*PKD1*
gRNA_2	c.1202-9G>A	TTCCCACCAGTCTCCAGCGG	human	*PKD1*
gRNA_3	c.1261C>T	CAGGCAGTAGCAGTGCCCGT	human	*PKD1*
gRNA_4	c.1816C>T	GCAGCCACAGCTGGGCGGGC	human	*PKD1*
gRNA_5	c.1831C>T	GGAGCCAGTACACCTGCAGC	human	*PKD1*
gRNA_6	c.2839C>T	TCCCTACAGTACACGGGCCT	human	*PKD1*
gRNA_7	c.3037G>A	TAACCATGGAGCGGATGAAC	human	*PKD1*
gRNA_8	c.3490G>A	GACTTCAGGGACGGCTCCCC	human	*PKD1*
gRNA_9	c.3955G>A	TCACCAGGAACCCGGCCCAC	human	*PKD1*
gRNA_10	c.3984G>A	TTCGACTGAACCTTCGGGGA	human	*PKD1*
gRNA_11	c.4322A>G	CAAGACAGGAGCCTGGGTCT	human	*PKD1*
gRNA_12	c.4906C>T	CCACCTACAGCCCCTCTATG	human	*PKD1*
gRNA_13	c.5086C>T	CAGCTACACATGGTAGGTGC	human	*PKD1*
gRNA_14	c.5477G>A	CTTTTAGGGGCAGCTGGCCA	human	*PKD1*
gRNA_15	c.6011A>G	CCAGGCGCAGGCGACCCGAG	human	*PKD1*
gRNA_16	c.6031C>T	CTTCTACAGCGAGAAGTACC	human	*PKD1*
gRNA_17	c.6406C>T	CGTGGCCTACGCCACGAAGA	human	*PKD1*
gRNA_18	c.6472C>T	CACCTACAGGGGCAGGACCA	human	*PKD1*
gRNA_19	c.6487C>T	ATCACCGCATCAGCACCTGC	human	*PKD1*
gRNA_20	c.6658C>T	AGCCACGGCAGCACCAGCCG	human	*PKD1*
gRNA_21	c.6743A>G	TCACACTGGCCTGGATGCTC	human	*PKD1*
gRNA_22	c.6916-9G>A	CTCAGCCTGCAGAGGGAGGC	human	*PKD1*
gRNA_23	c.7244T>C	TGCACGCACGTTCAGCAACA	human	*PKD1*
gRNA_24	c.7271C>T	GCCCATGGATGTGGTGGTCT	human	*PKD1*
gRNA_25	c.7915C>T	GGGCTCAGTGCTGCCGCTCG	human	*PKD1*
gRNA_26	c.7949T>C	GAGACTCCGGTGTCCCTGAG	human	*PKD1*
gRNA_27	c.7984C>T	CTGCTAGATGTCATCCACAG	human	*PKD1*
gRNA_28	c.8311G>A	CAACAAGGAGCCCCTGACGC	human	*PKD1*
gRNA_29	c.8381T>C	AGCCTGCCGTGCTATGGCGG	human	*PKD1*
gRNA_30	c.8558T>C	ATGGCATCCCAGACACAGGC	human	*PKD1*
gRNA_31	c.8593C>T	CAGCCACTCGATGGGGATCT	human	*PKD1*
gRNA_32	c.8929A>G	AAGGCGTAGGGCCGGTGGTC	human	*PKD1*
gRNA_33	c.9335G>A	CTTTCTATGGGCAGCGGGGC	human	*PKD1*
gRNA_34	c.9340C>T	CCGCTACCCACAGAAAGGGA	human	*PKD1*
gRNA_35	c.9547C>T	CCACACTCAGATCTTCCACA	human	*PKD1*
gRNA_36	c.9851G>A	CCTGCTACGTTCTCCTCATC	human	*PKD1*
gRNA_37	c.10290G>A	CCATTGTAGGTAGCAATCTG	human	*PKD1*
gRNA_38	c.10945C>T	CCGTGGGATGGCCGTACGCG	human	*PKD1*
gRNA_39	c.10951G>A	CCACAGCTTTGCACTCTTCC	human	*PKD1*
gRNA_40	c.11156+3A>G	GCCCGCACCGCGTGATGGCC	human	*PKD1*
gRNA_41	c.11384G>A	GACGTAGGCCTATTCAGCGC	human	*PKD1*
gRNA_42	c.12010C>T	TGCTAGGCAGCCTGCGGACG	human	*PKD1*
gRNA_43	c.12061C>T	CAGAGCTCAGCATAATGTCT	human	*PKD1*
gRNA_44	c.12124C>T	CAGCTAGGCGTAGGCTACCC	human	*PKD1*
gRNA_45	c.6646C>T_1	AGCCATGGCACCACCAGCTG	mouse	*Pkd1*
gRNA_46	c.6646C>T_2	CAGCCATGGCACCACCAGCT	mouse	*Pkd1*
gRNA_47	c.6646C>T_3	CCAGCCATGGCACCACCAGC	mouse	*Pkd1*
gRNA_48	c.6646C>T_4	GCCATGGCACCACCAGCTGG	mouse	*Pkd1*
